# Factors controlling the oxygen isotopic composition of lacustrine authigenic carbonates in Western China: implications for paleoclimate reconstructions

**DOI:** 10.1038/s41598-020-73422-4

**Published:** 2020-10-01

**Authors:** Huashu Li, Xingqi Liu, Aradhna Tripati, Shengnan Feng, Ben Elliott, Chloe Whicker, Alexandrea Arnold, Anne Marie Kelley

**Affiliations:** 1 College of Resource Environment and Tourism, Capital Normal University, 105 West-Third-Ring North Road, Haidian District, Beijing, 100048 People’s Republic of China; 2 Department of Earth, Planetary, and Space Sciences, Department of Atmospheric and Oceanic Sciences, Institute of the Environment and Sustainability, Center for Diverse Leadership in Science, University of California, Los Angeles, CA 90095 USA; 3 European Institute of Marine Sciences (IUEM), IFREMER, Laboratoire Géophysique et enregistrement Sédimentaire, Université de Brest, UMR 6538, Domaines Océaniques, Rue Dumont D’Urville, 29280 Plouzané, France

**Keywords:** Limnology, Climate sciences, Climate change, Limnology, Palaeoclimate

## Abstract

In the carbonate-water system, at equilibrium, the oxygen isotopic composition of carbonate is dependent not only on the temperature but also on the isotopic composition of host water in which the carbonate is formed. In this study, lake surface sediment and water samples were collected from 33 terminal lakes in Western China to evaluate controls on the oxygen isotopic composition of lacustrine authigenic carbonates (δ^18^O_carb_) and its spatial distribution. Our results show that water oxygen isotopic composition (δ^18^O_water_) rather than lake summer water temperature (T_water_), is the main determinant of δ^18^O_carb_, irrespective of whether oxygen isotope equilibrium is achieved. There are significant linear correlations between δ^18^O_carb_ and elevation, as well as that between δ^18^O_carb_ and latitude for lakes located on the Tibetan Plateau. In Western China, the spatial distribution of δ^18^O_carb_ is consistent with that of δ^18^O_water_, and is ultimately controlled by the isotopic composition of local precipitation (δ^18^O_precipitation_) that depends on the source of water vapor. Therefore, changes in δ^18^O_carb_ can be predominantly interpreted as variations of δ^18^O_water_, which in turn represent changes in δ^18^O_precipitation_ for paleoclimate reconstructions in this region, and may be relevant to studies of other areas.

The carbonate oxygen isotope geothermometer has been one of the most important tools for reconstructing paleotemperatures since it was established by Urey^1^ and McCrea^2^. In systems that are in thermodynamic equilibrium, an increase in temperature with increased atomic vibration frequency leads to decrease in isotopic fractionation between the reactant water and the carbonate mineral that precipitates from it^3–5^. Accordingly, a number of empirical relationships between carbonate oxygen isotopic composition and temperature have been established^5–8^, and are widely applied to lakes in paleoclimate studies to reconstruct water temperature^9–11^.

Our work on oxygen isotope systematics in modern lake carbonates is motivated by three issues. First, the fundamental precondition to applying an isotopic geothermometer is that carbonate precipitating from water is in oxygen isotope equilibrium^1,12^. The assumption of equilibrium has not been tested in detail in modern lacustrine authigenic carbonates and there are discrepancies relating to equilibrium fractionation factors and kinetic fractionation processes among theoretical calculations, experimental measurements, and modern natural observations^13,14^. In slowly precipitating experiments from Kim and O'Neil^7^, the authors judged the smallest fractionation factors to be the best representations of equilibrium fractionation factors, despite the fact that fractionation factors increased with increasing initial concentrations of metal ion and bicarbonate at a certain temperature. However, Zeebe^15^ argued that the observed effects by Kim and O'Neil^7^ were actually equilibrium fractionations expressed at different solution pH values. Based on Devils Hole calcite deposition, which is considered to be the most likely to approach to true thermodynamic equilibrium, Coplen^16^ considered that formerly adopted oxygen isotopic fractionation factors might be underestimated when used in natural samples because of the restriction of experimental conditions and theoretical calculation methods. Analysis of clumped isotopes in a series of samples led the authors to conclude that most carbonates precipitate out of equilibrium^17^. A study by Watkins et al*.*^18^ first isolated the kinetic oxygen isotope effects occurring at the calcite-water interface and analyzed the effects of changing calcite precipitation rates and water pH. Recent studies from Devriendt et al*.*^19^ and Guo et al*.*^20^ also confirmed that different solution pH values, crystal growth rates, and mass-dependent processes might lead to disequilibrium fractionations. In addition, most laboratory experiments and theoretical calculations are based on limiting multiple external conditions with only one variable; however, natural settings are more complex. This may result in inconsistencies of calculated oxygen isotope fractionation factors among different laboratories, as well as that between natural records and laboratory experiments^18,21^. Co-variation between lake carbonate oxygen and carbon isotopes have been used to identify lake systems where kinetics dominate^9,22^. Therefore, an accurate determination of whether equilibrium fractionation occurs, and an understanding of the processes that control kinetic fractionation are essential for the accurate interpretation of carbonate oxygen isotopic composition as an environmental proxy.

Second, the oxygen isotopic composition of lake water for geologic samples is often difficult to constrain. In many paleoclimate reconstructions, where it is often assumed carbonate precipitation is in near equilibrium, there are often no independent constraints on either temperature or water isotopes^5^. Prior work relies on an assumption of the lake water oxygen isotopes or numerical modeling^11,23^. In addition, some field studies show that the oxygen isotopic compositions of lacustrine carbonates are largely influenced by rainfall or local precipitation/evaporation balance, rather than temperature changes^24,25^.

Third, lakes play a pivotal role as recorders of climate change, and lacustrine authigenic carbonates are an ideal material for isotope analyses utilized in a number of studies^5,26,27^. However, there are few studies that have examined the modern oxygen isotopic systematics of authigenic carbonates in lakes; most have focused on investigations of paleoenvironments^5,28–30^. Therefore, an understanding of whether lacustrine authigenic carbonates can achieve oxygen isotope equilibrium when they were precipitated in modern natural lake settings, and understanding of which factors, such as water temperature, precipitation, lake elevation, latitude or longitude, could be reflected by the oxygen isotopic composition of lacustrine authigenic carbonates collected from a certain region is essential for accurate interpretation for paleoclimate reconstructions.

Western China is considered to be a semi-arid and arid region, characterized by low, uneven rainfall and high evaporation^31^. There are a large number of natural lakes distributed in Western China, which account for nearly 58.3% of the number and 66.9% of the area of all Chinese lakes^32^. Due to the influence and limitations of complex geological formations and natural environments, most of the lakes are terminal lakes with relatively high salinities^33–35^. Based on the large differences of geographical locations, basin topography, local climates, and water hydrology conditions, these lakes are sensitive to environmental changes, especially to variations in temperature and humidity^36^.

In this study, we present measurements from modern lacustrine authigenic carbonates and lake surface water samples collected from thirty-three terminal lakes in Western China ranging in elevation and temperature (Fig. 1). We also compare our results with a compilation of more than five hundred oxygen isotope measurements of different types of carbonates from published papers. The aims of this study are: (1) to investigate the relationships between the oxygen isotopic composition of lacustrine authigenic carbonate and that of respective host waters, as well as the relationships between the carbonate oxygen isotope fractionation factor and temperature in natural lake settings; (2) to explore the spatial variations and dominant factors influencing the oxygen isotopic composition of lacustrine authigenic carbonate in Western China, and to provide a framework that can be used for lacustrine paleoclimate reconstructions.

**Figure 1:**
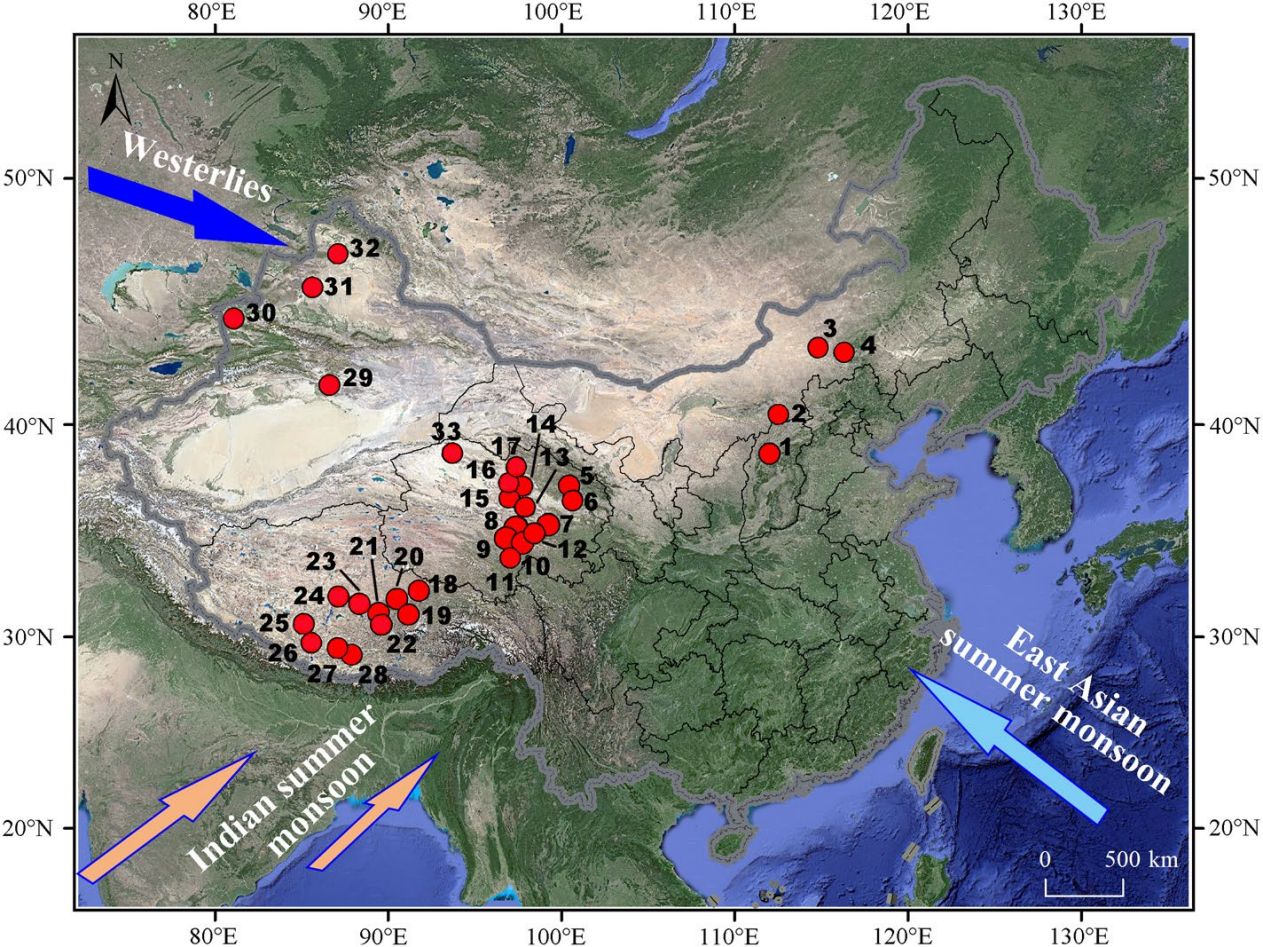
The locations of thirty-three lakes where lacustrine sediments and water samples were collected in China. Numbers and lake names are listed in Supplementary Table S1 online. The software BIGEMAP (https://www.bigemap.com) was used in this study to download the satellite imagery from Google Earth (Map data: Google, Maxar Technologies). The spatial location, the data layer creation and “shape” vector format file generation were performed in software ArcGIS 10.2.

## Results

### Summer water surface temperature calculation

Logged Mean Summer Water Temperature (T_LMSW_) from June to August was calculated using the data recorded by the on-site data loggers retrieved from twelve lakes (Supplementary Table S1 online). We derived a regression based on the relationship between Midday Temporal Water Temperature (T_MTW_), measured manually using a mercurial thermometer in the field, and the T_LMSW_ from the loggers that were able to be retrieved from the lakes:

1 $${\text{T}}_{{{\text{LMSW}}}} = { 1}.{19} \pm 0.0{\text{9T}}_{{{\text{MTW}}}} - {4}.{43} \pm {1}.{61}\left( {{\text{n }} = { 12},{\text{ r }} = \, 0.{97}, \, P \, < \, 0.000{1}} \right)$$

**Figure 2:**
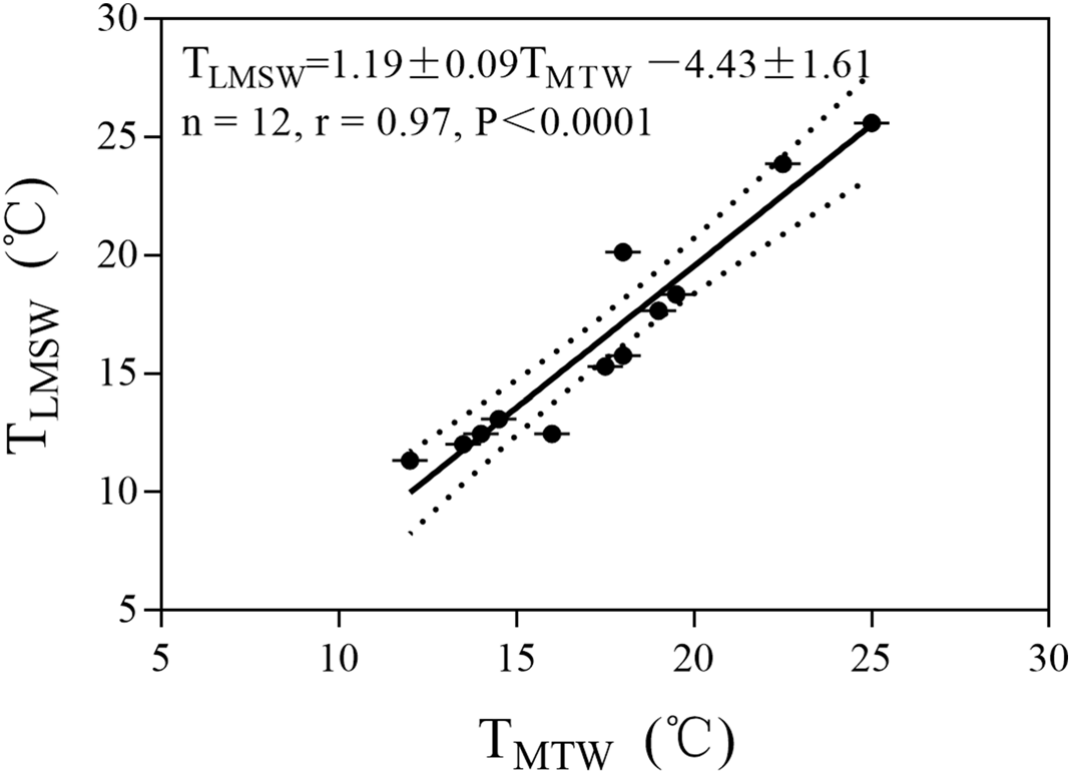
Regression between Midday Temporal Water Temperature (T_MTW_) and Logged Mean Summer Water Temperature (T_LMSW_) showing a significant correlation (*P* < 0.0001). Calculated Mean Summer Water Temperature (T_CMSW_) was calculated using the regression formula: T_CMSW_ = 1.19 ± 0.09T_MTW_ − 4.43 ± 1.61 (r = 0.97, *P* < 0.0001). The dotted lines show 95% confidence intervals.

The T_LMSW_ is positively correlated to the T_MTW_ (Fig. 2). For 21 sites where water temperature loggers were lost in the field, we determined the Calculated Mean Summer Water Temperature (T_CMSW_) by applying Eq. (1) to the T_MTW_ values for the lakes without loggers. Thus, in this study, lake summer water surface temperature (T_water_) is either T_LMSW_ (lakes with loggers) or T_CMSW_ (lakes without loggers). T_water_ ranged from 9.8 to 25.6 °C (Supplementary Table S1 online).

### Lake surface water and surface sediment information

The salinity of the studied thirty-three lake surface waters ranged from 354.18 to 87,991.23 mg/L. The pH values ranged from 7.89 to 9.81. The saturation index (SI) value of calcite, aragonite, or dolomite exceeds 0 for each sample (Supplementary Table S2 online). Although there is no relevant data to calculate the SI value of monohydrocalcite, we assumed the waters were supersaturated in monohydrocalcite in the cases where rapid deposition occurred. The oxygen and hydrogen isotopic composition of lake surface water (δ^18^O_water_ and δD_water_) ranged from − 8.82 to 5.65‰ (VSMOW) and from − 79.12 to 11.22‰ (VSMOW), respectively (Supplementary Table S2 online).

In absence of detrital and biogenic carbonate, the fine sieved carbonate smaller than 45 μm can be characterized as authigenic carbonate, which is chemically precipitated in lake water^37^. X-ray powder diffraction (XRD) analyses show that there are nine pure calcite samples and twenty-four mixed mineralogy samples in this study (Supplementary Table S3 and Fig. S1 online). The oxygen isotopic composition of lacustrine authigenic carbonate (δ^18^O_carb_) was calculated based on a stable isotope mixing model. δ^18^O_carb_ spanned a relatively large range with a minimum of − 9.61 ‰ (VPDB) and a maximum of 3.77‰ (VPDB) between lakes. The oxygen isotope fractionation factor between carbonate and water (1000lnα_(carb−water)_) ranged from 22.38 to 32.71. Carbon isotopic composition of lacustrine authigenic carbonate (δ^13^C_carb_) ranged from − 4.06 to 4.97‰ (VPDB) (Supplementary Table S3 online).

### Relationships between 1000lnα_(carb−water)_, T_water_, δ^18^O_carb_ and δ^18^O_water_

**Figure 3:**
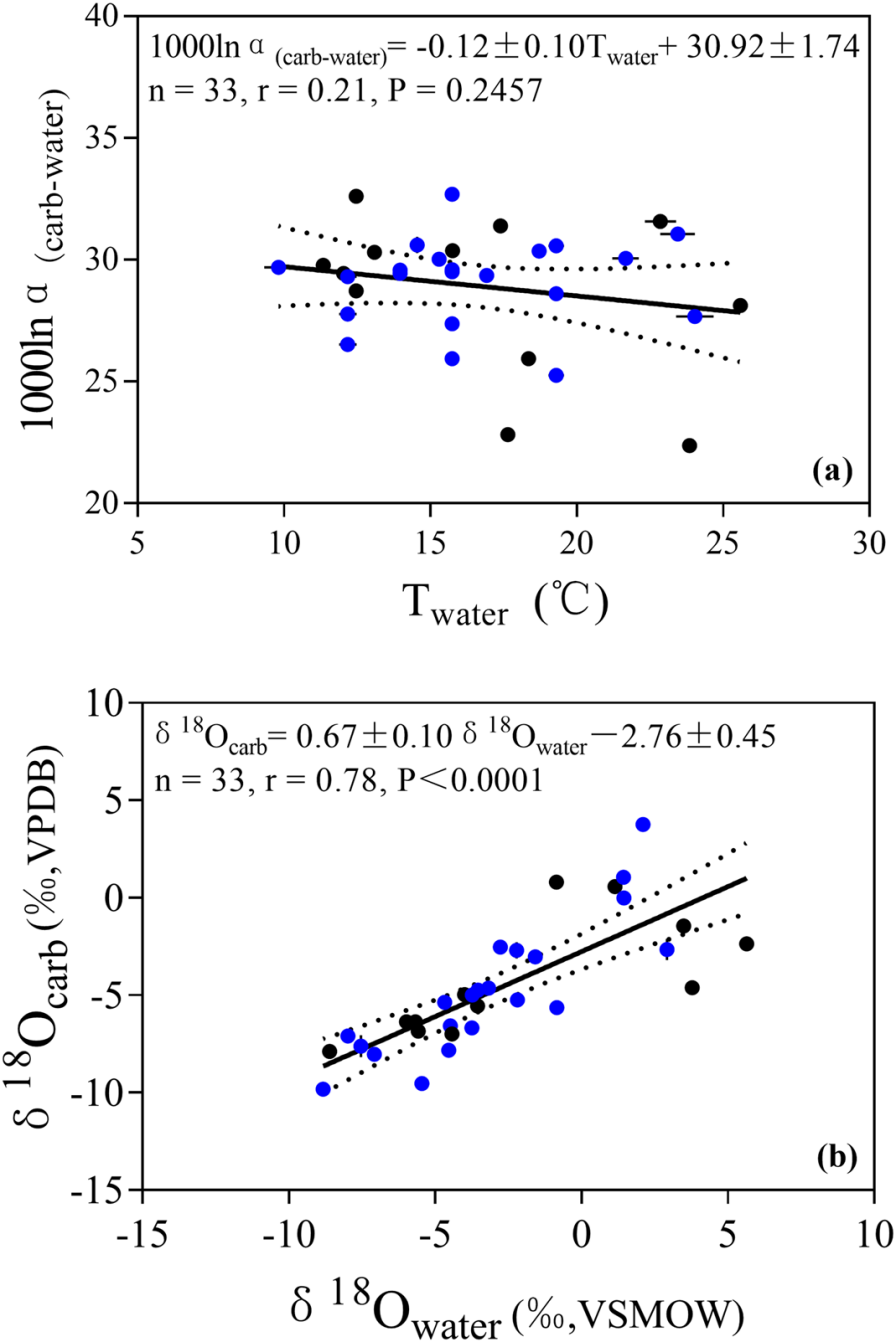
Comparison of (**a**) T_water_ and 1000lnα_(carb−water)_; (**b**) δ^18^O_water_ and δ^18^O_carb_ values of the thirty-three lakes in this study. Black points refer to water temperatures directly recorded by on-site water temperature loggers (T_LMSW_). Blue points refer to water temperatures for sites without data loggers and are calculated using the Eq. (1) and are reported as Calculated Mean Summer Water Temperature (T_CMSW_). Similar results are obtained using both types of temperature data. Solid lines are least-square linear regression lines. Error bars show standard deviations in each sample. Dotted lines show 95% confidence intervals.

As shown in Fig. 3a, there is no statistically significant correlation between 1000lnα_(carb−water)_ and T_water_:

2 $${1}000{\ln}\alpha_{{({\text{carb}} - {\text{water}})}} = \, - \, 0.{12} \pm \, 0.{1}0{\text{T}}_{{{\text{water}}}} + { 3}0.{92} \pm { 1}.{74}\left( {{\text{n }} = { 33},{\text{ r }} = \, 0.{21},{\text{ P }} = \, 0.{2457}} \right)$$

All points are scattered on both sides of the fitted line, where 18 points distribute out of the 95% confidence interval. Thus, an initial inspection indicates the 1000lnα_(carb−water)_ values cannot be used to infer lake summer water temperature.

The comparison of δ^18^O_carb_ with δ^18^O_water_ shows a significant correlation:

3 $$\delta^{18} {\text{O}}_{{{\text{carb}}}} = 0.67 \pm 0.10\delta^{18} {\text{O}}_{{{\text{water}}}} - \, 2.76 \pm 0.45\left( {{\text{n }} = \, 33,{\text{ r }} = \, 0.78, \, P < 0.0001} \right)$$

In general, δ^18^O_carb_ exhibit a strong positive correlation with δ^18^O_water_, with higher δ^18^O_water_ corresponding with more enriched δ^18^O_carb_ (Fig. 3b).

**Figure 4:**
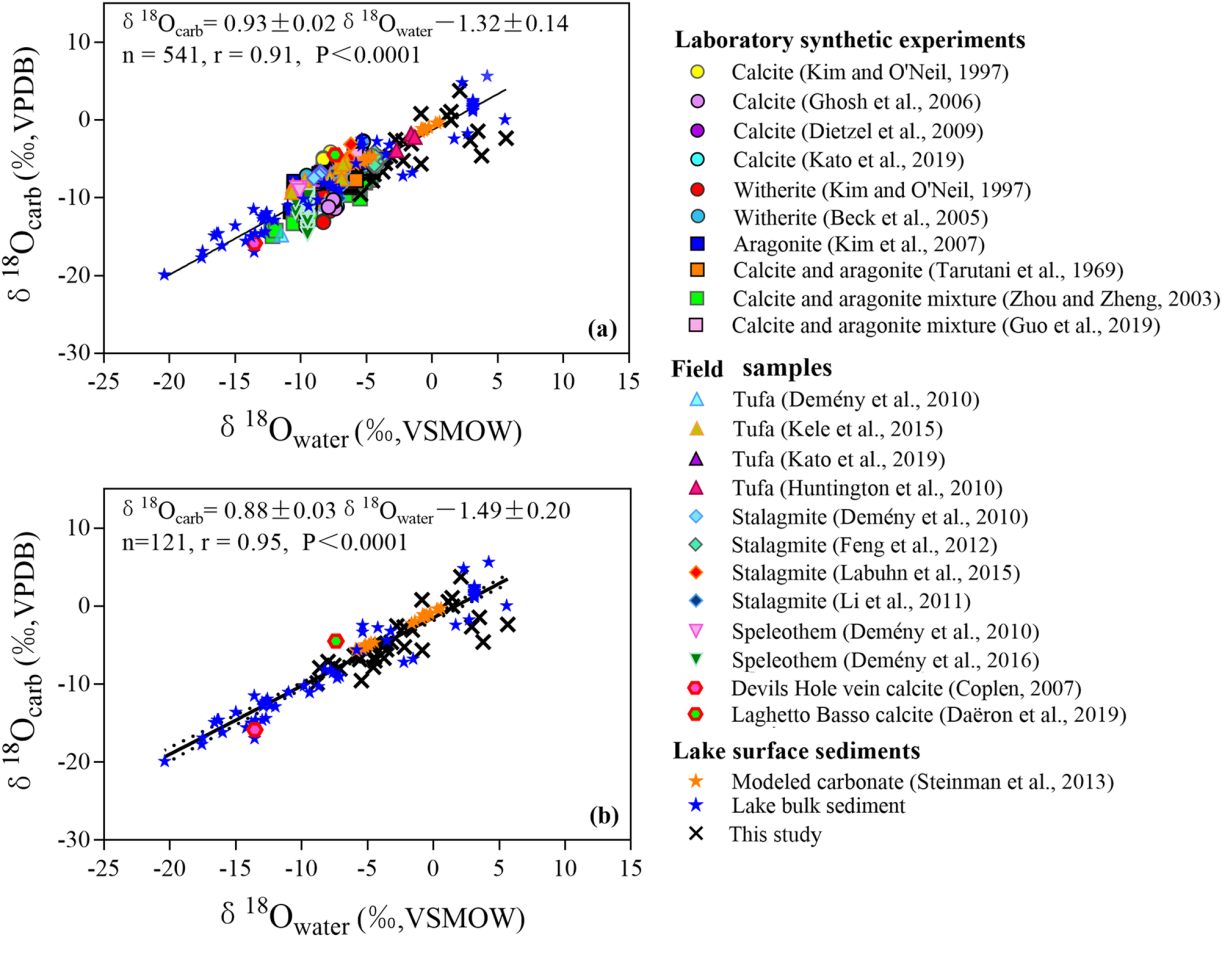
Relationship between oxygen isotopic composition of carbonate and host waters in (**a**) a combination of isotope data collected from published papers and thirty-three lakes, and in (**b**) lake surface sediments. Black crosses are data for thirty-three lake surface water samples analyzed in this study. Also shown are data for laboratory synthetic carbonates from experiments, lake surface sediment samples, and other field-collected samples compiled from published papers (Supplementary Table S4 online). The black solid line is the least-square linear regression line through all data. Dotted line shows 95% confidence intervals.

We collated 508 carbonate and water isotope data from published papers (Supplementary Table S4 online). The combination of oxygen isotopic composition collected from thirty-three lakes and published papers covers a large gradient: δ^18^O_water_ ranged from − 20.4 to 5.65‰ (VSMOW) and δ^18^O_carb_ ranged from − 19.9 to 5.65‰ (VPDB). There is a statistically significant linear correlation between δ^18^O_carb_ and δ^18^O_water_ from combined isotope data in different sections (Fig. 4a):

4 $$\delta^{{{18}}} {\text{O}}_{{{\text{carb}}}} = \, 0.{93} \pm \, 0.0{2}\delta^{{{18}}} {\text{O}}_{{{\text{water}}}} - {1}.{32} \pm \, 0.{14}\left( {{\text{n }} = { 541},{\text{ r }} = \, 0.{91}, \, P \, < \, 0.000{1}} \right)$$

The linear regression between δ^18^O_carb_ and δ^18^O_water_ from combined lake surface sediment samples (Fig. 4b) is:

5 $$\delta^{{{18}}} {\text{O}}_{{{\text{carb}}}} = \, 0.{88} \pm \, 0.0{3}\delta^{{{18}}} {\text{O}}_{{{\text{water}}}} - { 1}.{49} \pm \, 0.{2}0\left( {{\text{n }} = { 121},{\text{ r }} = \, 0.{95}, \, P \, < \, 0.000{1}} \right)$$

### Spatial distribution of δ^18^O_carb_

**Figure 5:**
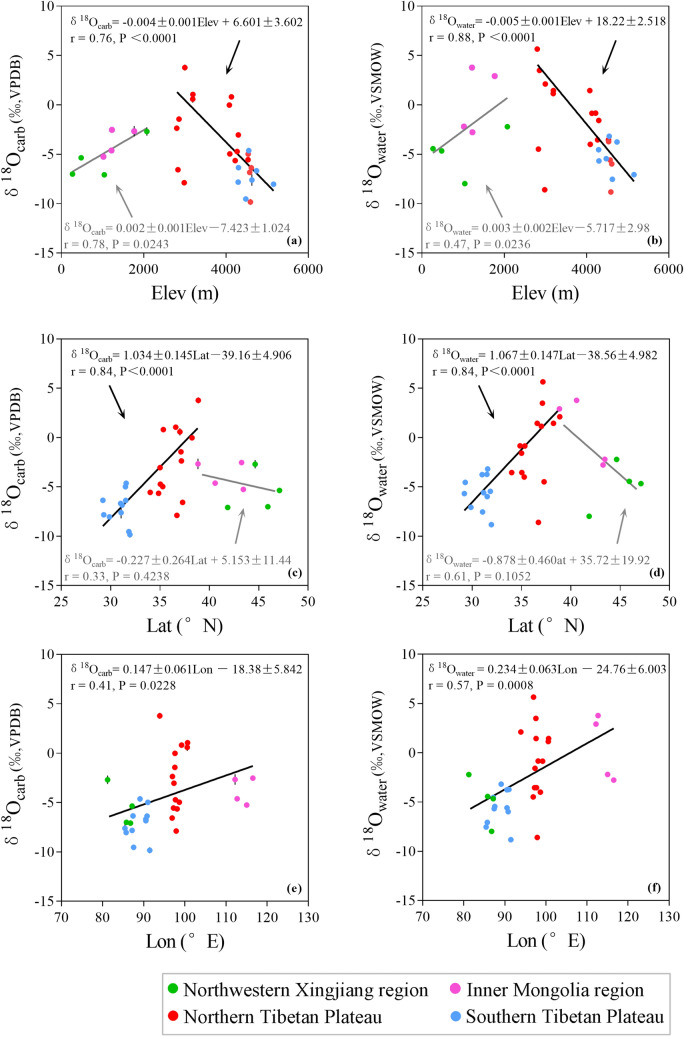
Relationship between δ^18^O_carb_ and (**a**) lake elevation (Elev); (**c**) lake latitude (Lat); and (**e**) lake longitude (Lon). Relationship between δ^18^O_water_ and (**b**) Elev; (**d**) Lat; and (**f**) Lon at the thirty-three sites sampled in this study^64^. Red points denote lakes located on the Northern Tibetan Plateau, light blue points denote lakes located on the Southern Tibetan Plateau, green points denote lakes located in the Northwestern Xinjiang region, pink points denote lakes locate at the Inner Mongolia region.

As shown in Fig. 5a, the variations of δ^18^O_carb_ show a strong dependence (r = 0.76, *P* < 0.0001) on lake elevation (Elev) on the Tibetan Plateau. But the Elev/δ^18^O_carb_ coefficient is not very significant (r = 0.78, *P* = 0.0243) for the Northwestern Xinjiang and Inner Mongolia regions where lake elevation below 3000 m. On the Tibetan Plateau, there is a positive linear correlation between lake latitude and δ^18^O_carb_ (r = 0.84, *P* < 0.0001) (Fig. 5c). But it displays inverse spatial variations of δ^18^O_carb_ for the Northwestern Xinjiang and Inner Mongolia regions. The linear correlation exists between lake longitude and δ^18^O_carb_ is not very statistically significant (r = 0.41, *P* = 0.0228) in Western China (Fig. 5e). In general, the spatial distributions of δ^18^O_carb_ are consistent with that of δ^18^O_water_ in Western China, and the four regions in Western China can be clearly distinguished by variance in δ^18^O_carb_ and δ^18^O_water_ values (Fig. 5).

## Discussion

Two critical factors controlling the oxygen isotopic composition of carbonate mineral are the temperature of carbonate formation and the oxygen isotopic composition of the solution from which carbonate minerals precipitate^5^. In this section, we discuss the effects of these two factors respectively and spatial distributions of the oxygen isotopic composition of lacustrine carbonates in Western China.

In this study, the carbonate samples provide integrated climate signals cover several years as the sedimentation rate of surface sediment ranges from 0.01 to 0.3 cm/yr throughout our sampling locations^37–41^. In general, lacustrine authigenic carbonates precipitate in summer, when the carbonate saturation of lake water peaks and carbonate solubility is simultaneously depressed in the epilimnion^5,9,42,43^. Variations in water temperatures averaged over several summers in recent years usually less than ± 1 ~ 2 °C at a certain lake, especially for the lakes located on the Tibetan Plateau^44,45^. Therefore, the measured summer water temperatures could represent the temperatures when authigenic carbonate samples were precipitated. In large closed lake systems, variations in water isotope composition caused by precipitation or evaporation are usually homogenized by buffering of large lake volume^5,24^. Therefore, a large lake with long water residence time could ‘average out’ short-term changes in isotope compositions and instead reflects relatively long-term isotope compositions under similar climate and hydrological conditions^5,46,47^. As the size of sampled thirty-three lakes is relatively large, and the relative humidity in Western China has not changed greatly in recent years^48,49^, variations in water isotope values during the course of one or several summers may not significant^50^. Because we do not have longitudinal data on lake water isotope values at our lakes, we assume that the measured isotope values of water samples that collected at the lake center and at the same time with sediment samples could be considered as long-term average compositions during the summer when authigenic carbonates were precipitated.

**Figure 6:**
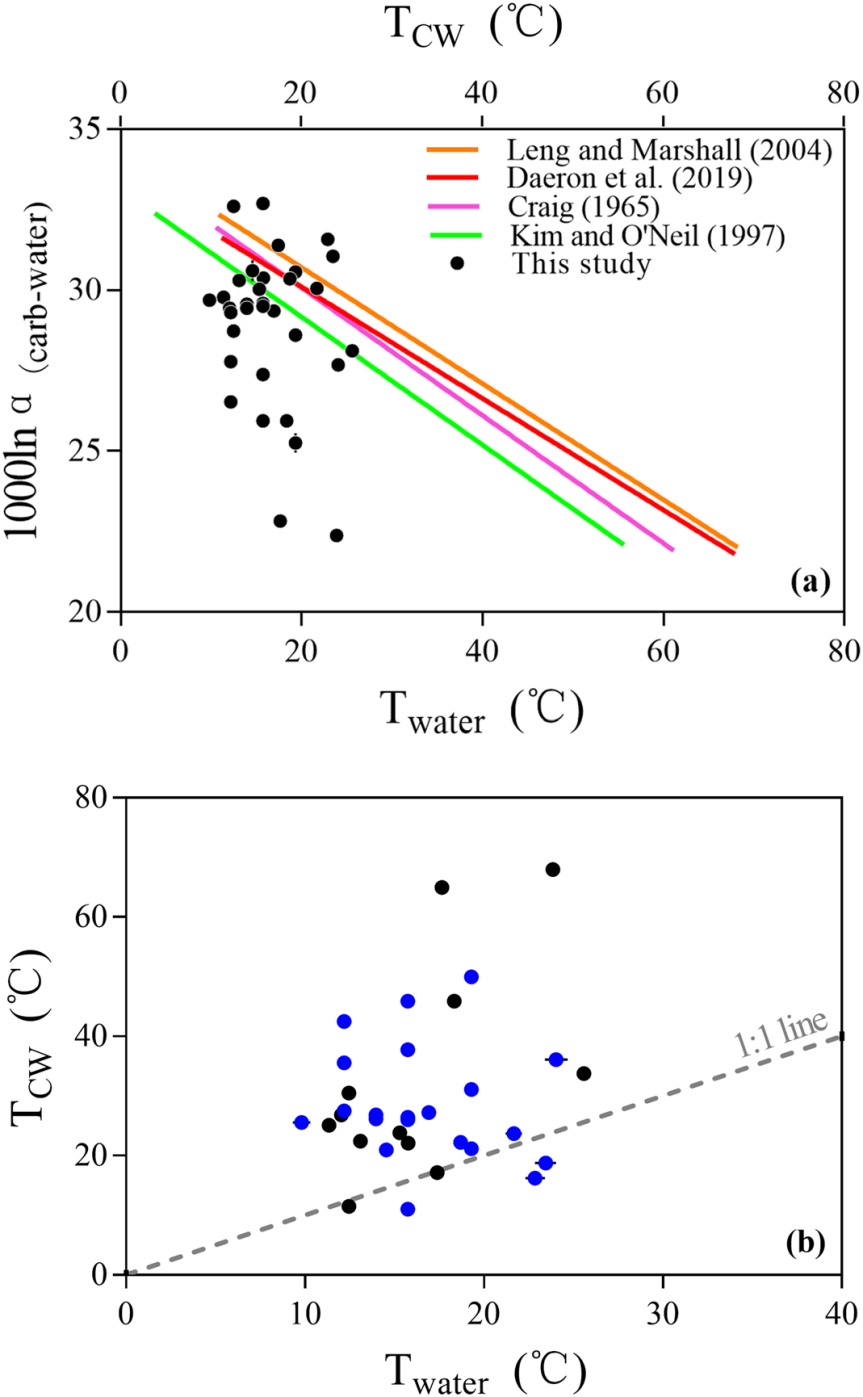
Relationship between (**a**) lacustrine authigenic carbonate oxygen isotope fractionation factors (1000lnα_(carb−water)_) and lake summer water surface temperature (T_water_) and modeled water temperatures (T_CW_) by applying several published temperature calibrations^5–7,17^; and (**b**) lake summer water surface temperature (T_water_) and modeled water temperatures (T_CW_) using the Daëron et al*.*^17^ calibration indicating temperature is not the dominant control on the δ^18^O of lacustrine authigenic carbonates sampled in Western China. Circles are data from thirty-three samples collected for this study. Black points refer to water temperatures directly recorded by on-site water temperature loggers (T_LMSW_). Blue points refer to water temperatures for sites without data loggers and are calculated using the Eq. (1) and are reported as Calculated Mean Summer Water Temperature (T_CMSW_). Similar results are obtained for both types of temperature data. Solid lines are least-square linear regression lines. Dashed line represents 1:1 (i.e. no difference) relationship.

For equilibrium carbonate precipitation, oxygen isotope fractionation is directly controlled by thermodynamics, and the isotope fractionation factor is a function of temperature^1, 2^. However, there is no statistically significant correlation between the 1000lnα_(carb−water)_ and water temperatures in natural lake settings in this study (Fig. 3a). Since calcites from the Devils Hole and Laghetto Basso are considered to be most representative of true thermodynamic equilibrium, we further compared our results with the equilibrium baseline defined by Daëron et al*.*^17^ and other published temperature dependent oxygen isotope equilibrium fractionations^5–7^. We modeled water temperatures (T_CW_) by applying published temperature calibrations to the 1000lnα_(carb−water)_ values of our lakes, and compared modeled T_CW_ with independently measured T_water_. As shown in Fig. 6a, the modeled T_CW_ ranges from 3.8 to 68.2 °C, which is far beyond T_water_ (9.8–25.6 °C) of our lakes. The slope and intercept of 1000lnα_(carb−water)_-T_CW_ regression lines are lower than that of 1000lnα_(carb−water)_-T_water_ regression line (Fig. 6a). The discrepancy between our results and published calibrations is salient as shown in Fig. 6b, in which most of modeled T_CW_ using the Daëron et al*.*^17^ calibration are higher than the T_water_ and no statistically significant correlation existed between T_CW_ and T_water_ (*P* = 0.2199). This suggests that factors other than temperature are contributing to oxygen isotope fractionation observed in lacustrine authigenic carbonates in this study. The discrepancies between our results and previous studies may be attributed to carbonate disequilibrium precipitation.

Lacustrine authigenic carbonates form in a mixture of dissolved inorganic carbon (DIC) species in lake water. At equilibrium, the oxygen isotopic composition of precipitated carbonate closely approximates that of DIC at a certain temperature^18,51^. As lake water is the largest reservoir of oxygen isotopes for DIC species and precipitated carbonates, we employed a mass balance calculation^13,52^ to calculate the expected oxygen isotope fractionation factor between DIC and water $(1000{\ln}{\upalpha}_{\left({\text{DIC}}{-}{\text{H}}_{2}{\text{O}}\right)})$ under equilibrium fractionation, in order to investigate whether equilibrium oxygen isotope fractionation are achieved in 33 lakes in Western China. For the calculation of expected equilibrium $(1000{\ln}{\upalpha}_{\left({\text{DIC}}{-}{\text{H}}_{2}{\text{O}}\right)})$ values, we neglect CO_2(aq)_ because there should not be a substantial influence of CO_2_ degassing on the DIC pool in natural lake settings^53^. The mass balance equation is:

6 $${1}000{\ln}\alpha_{{({\text{DIC}} - {\text{H}}_{2}^{ \cdot } {\text{O)}}}} = X_{{{\text{HCO}}_{3}^{ - \cdot } }} \left( {{1}000{\ln}\alpha_{{({\text{HCO}}_{3}^{ - \cdot } - {\text{H}}_{2}^{ \cdot } {\text{O)}}}} } \right) \, + X_{{{\text{CO}}_{3}^{2 - \cdot } }} \left( {{1}000{\ln}\alpha_{{{\text{(CO}}_{3}^{2 - \cdot } - {\text{H}}_{2}^{ \cdot } {\text{O}})}} } \right)$$

where *X* denotes the molar fractionation of the DIC species which is determined by PHREEQC v. 2.18.00. software program^54^, and 1000lnα*i* denotes the individual fractionation factor between the DIC species and lake water reported by Beck et al*.*^51^ at a certain summer water temperature in each lake.

We compared our experimental 1000lnα_(carb−water)_ values to expected equilibrium $(1000{\ln}{\upalpha}_{\left({\text{DIC}}{-}{\text{H}}_{2}{\text{O}}\right)})$ values (Supplementary Fig. S2 online). There is no statistical linear correlation between the experimental 1000lnα_(carb−water)_ values and the expected equilibrium $(1000{\ln}{\upalpha}_{\left({\text{DIC}}{-}{\text{H}}_{2}{\text{O}}\right)})$ values (r = 0.23, *P* = 0.2033). Most of the 1000lnα_(carb−water)_ values are lower than the expected equilibrium $(1000{\ln}{\upalpha}_{\left({\text{DIC}}{-}{\text{H}}_{2}{\text{O}}\right)})$ values (Supplementary Table S3 online). The offsets ranging from 0.19 to 9.25 indicate that the oxygen isotope values of precipitated lacustrine authigenic carbonates may not in equilibrium with lake waters in this study, and the temperature is not the primary control on carbonate δ^18^O values in natural lake settings.

Carbonate disequilibrium precipitation may be influenced by multiple mixed kinetic fractionation processes that originate from kinetic fractionations during the exchange of oxygen isotopes between water and DIC species, or between DIC species and carbonate, or a combination of both of these factors^18,55^. With respect to lakes, there are two main factors that can lead to the disequilibrium fractionation that we observe; these may be complicated by multiple environmental controls and processes at a given site.

First, the pH value of lake water is an important factor in carbonate isotope fractionation processes. The pH value of the solution determines the concentration of each DIC species, which in turn controls the relative proportions of DIC species participating in carbonate growth at a certain temperature^15,56,57^. The oxygen isotope fractionation between water and DIC, as well as that between DIC and precipitated carbonates, will decrease with increasing pH, as the dominant DIC species changes from CO_2(aq)_ to $\text{CO}^{2-}_{3}$^18,55^. Given that pH values differ between the lakes in this study, we evaluated the pH effect using a model from Watkins et al.^55^ (Supplementary Fig. S3 online). Although several of the data points can be explained by a combination of temperature and pH, however, more than half of the samples fall out of the range predicted by the model (Supplementary Fig. S3 online), indicating pH values of lake water are not the dominant factor controlling the oxygen isotopic composition of lacustrine authigenic carbonates at many of the sites in Western China.

Second, the oxygen isotope exchange between DIC species and water is a rate-limiting step for equilibrium^13,58^. High carbonate growth rates may result in kinetic fractionation of a different magnitude^21,56,59^. The slower the carbonate is formed, the more likely the isotope fractionation between water and carbonate is to be close to equilibrium. Authigenic carbonates are thought to form in lacustrine settings relatively rapidly^37,60^, especially for carbonates precipitated from saturated solutions (SI > 0) as in this study. Since the growth rates of carbonate samples in this study are, as with many carbonates, likely to be higher than that of slowly precipitated calcites collected from the Devils Hole and Laghetto Basso, the time for oxygen exchange between the DIC and water might be insufficient to attain the equilibrium fractionation^16,17^. In this case, the DIC species with ^16^O isotopologues tend to preferentially participate in oxygen isotopic exchange, leading to the oxygen isotope composition of formed carbonates being lighter than theoretical values^20,55^. Therefore, kinetic fractionations caused by pH effect and growth rate effect might lead to large deviations of fractionation factors between expected equilibrium and measured results in natural lake settings, resulting in the lack of a significant correlation between 1000lnα_(carb−water)_ and T_water_ in this study.

In order to compare the relationship between δ^18^O_carb_ and δ^18^O_water_, we compiled more than five hundred oxygen isotope values of different types of carbonates from published papers (Fig. 4 and Supplementary Table S4 online). Although some of the published isotope data were assumed to have reached oxygen isotopic equilibrium, for most of the data, it is unclear whether oxygen isotopic equilibrium was reached when carbonate precipitated from host water. As shown in Fig. 4, the linear correlation between δ^18^O_carb_ and δ^18^O_water_ values is significant both in all carbonate data (n = 541, r = 0.91, *P* < 0.0001) and in lake surface sediments (n = 121, r = 0.95, *P* < 0.0001). These results indicate that higher δ^18^O_water_ values correspond to higher δ^18^O_carb_ values, even though hydrologic conditions differ between records and may be complex, and oxygen isotope equilibrium is not necessarily attained during carbonate precipitation. The positive correlation between δ^18^O_water_ and δ^18^O_carb_ can be explained by the exchange of oxygen isotopes during the formation of carbonate^4^:

7 $$\left[ {{\text{C}}^{18} {\text{O}}_{2}^{16} {\text{O}}} \right]^{2 - } + {\text{ H}}_{2}^{16} {\text{O}} \rightleftarrows \left[ {{\text{C}}^{18} {\text{O}}^{16} {\text{O}}_{2} } \right]^{2 - } + {\text{ H}}_{2}^{18} {\text{O}}$$

8 $${\text{H}}_{2}^{18} {\text{O }} + \left[ {{\text{C}}^{18} {\text{O}}^{16} {\text{O}}_{2} } \right]^{2 - } + {\text{ Ca}}^{2 + } \rightleftarrows {\text{CaC}}^{18} {\text{O}}_{2}^{16} {\text{O}}_{{({\text{s}})}} \downarrow \, + {\text{ H}}_{2}^{16} {\text{O}}$$

where heavier ^18^O is transferred from reactant H_2_O to precipitated CaCO_3_ minerals.

Furthermore, δ^18^O_water_, water pH and T_water_ were used in boosted regression tree (BRT) analyses to produce models that could estimate contributions of lake water parameters to δ^18^O_carb_ for our sample sites. The BRT results estimate that δ^18^O_water_, pH, and T_water_ account for 80.1%, 12%, and 7.9% of the variance in δ^18^O_carb_, respectively (Supplementary Fig. S4 online). This demonstrates δ^18^O_water_ rather than T_water_ is the primary variable influence on δ^18^O_carb_ within lakes in Western China.

Based on these observations, we find that irrespective of whether isotope equilibrium is achieved in natural lacustrine settings, the δ^18^O values of lake water is the dominant factor governing carbonate δ^18^O values. In this case, it is necessary to investigate the controlling factors of variations of δ^18^O_water_ and find out what can be reflected by δ^18^O_carb_ in Western China.

**Figure 7:**
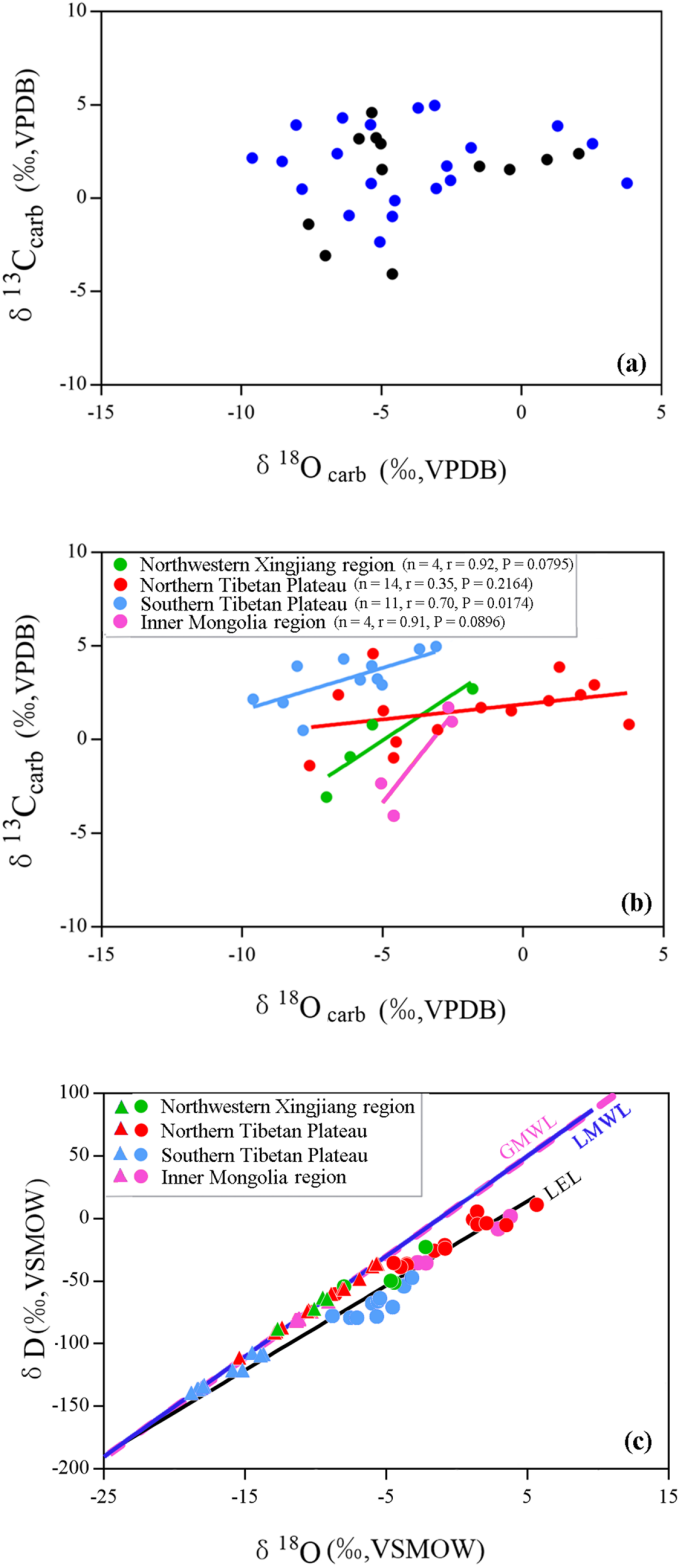
(**a**) Relationship between δ^18^O_carb_ and δ^13^C_carb_ in this study. No significant linear correlation between δ^18^O_carb_ and δ^13^C_carb_ (*P* = 0.4108) is observed for the thirty-three samples. Black points refer to water temperatures directly recorded by on-site water temperature loggers (T_LMSW_). Blue points refer to water temperatures for sites without data loggers and are calculated using the Eq. (1) and are reported as Calculated Mean Summer Water Temperature (T_CMSW_). (**b**) Relationship between δ^18^O_carb_ and δ^13^C_carb_ in this study. Samples are classified according to the position in which each lake located. Red points denote lakes located on the Northern Tibetan Plateau, light blue points denote lakes located on the Southern Tibetan Plateau, green points denote lakes located in the Northwestern Xinjiang region, pink points denote lakes locate at the Inner Mongolia region. The solid lines are least-square linear regression lines. (**c**) Oxygen and hydrogen isotopic composition of thirty-three water samples measured for this study^64^. Triangles are modern local precipitation in the study areas which are derived from the Online Isotopes in Precipitation Calculator^85^. Circles are lake surface water which coded in different colors according to the position in which each lake is located. The pink dashed line is the Global Meteoritic Water Line (GMWL)^86^. The dark blue solid line is Local Meteoritic Water Line (LMWL). The black solid line is Local Evaporation Line (LEL). The solid lines are least-square linear regression lines.

**Figure 8:**
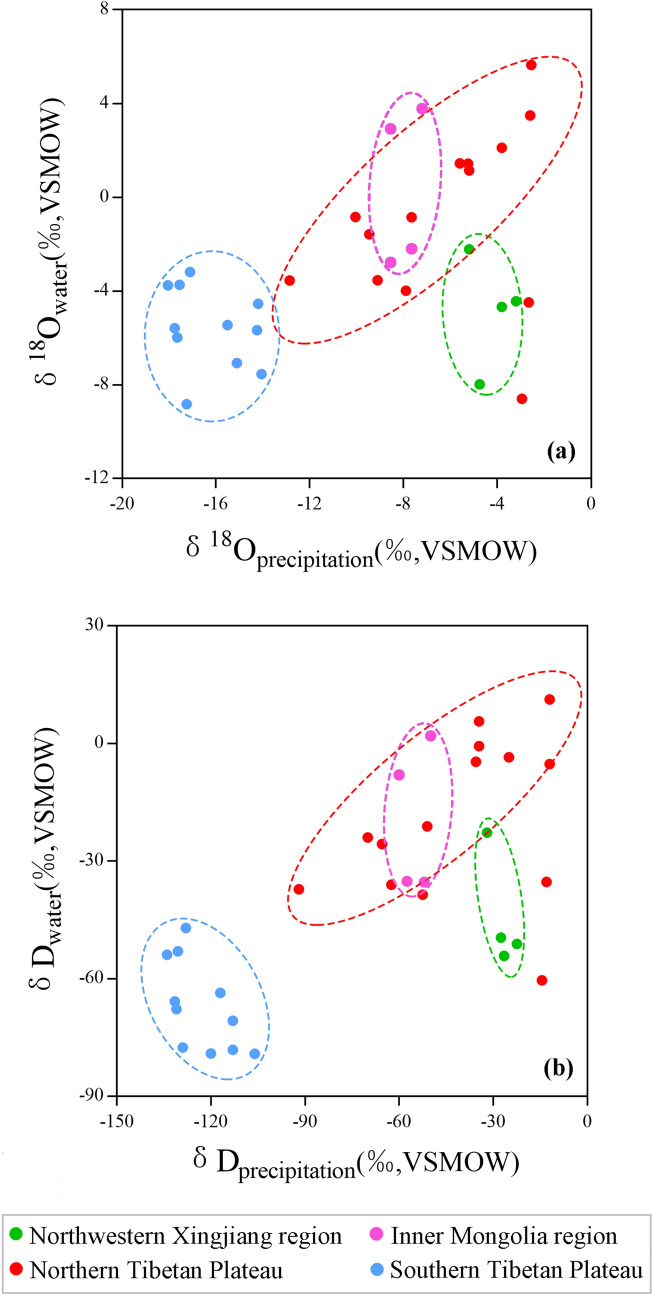
Relationship of isotope values between local precipitation and lake surface water at the thirty-three sites sampled in this study^64^. Red points denote lakes located on the Northern Tibetan Plateau, light blue points denote lakes located on the Southern Tibetan Plateau, green points denote lakes located in the Northwestern Xinjiang region, pink points denote lakes locate at the Inner Mongolia region.

As for the oxygen isotopic composition of lake water, it mainly depends on the changes of the isotopic composition of precipitation and local evaporation, in closed lake basins where rivers in the catchment are supplied by precipitation and no surface or groundwater output exists^61^. For terminal lakes, covariant trends between carbonate δ^18^O and δ^13^C reflect isotope enrichment caused by kinetic fractionation during evaporation^9,62,63^. As shown in Fig. 7a, there is no significant linear correlation (*P* = 0.4108) between δ^18^O_carb_ and δ^13^C_carb_ for the lakes in this study. But if we classify samples by the location of lakes, the correlations between δ^18^O_carb_ and δ^13^C_carb_ become more considerable. Linear correlation coefficients (r) range between 0.35 and 0.92 depending on the location of the lake (Fig. 7b). We infer that the influence of evaporation on δ^18^O_carb_ may be less than the influence of precipitation isotopic composition on δ^18^O_carb_ in Western China. This inference is also supported by spatial variations of lake water δD and δ^18^O at the 33 sites sampled for this study^64^. Feng et al*.*^64^ discussed the relationship between isotopic composition of lake water (δ^18^O_water_ and δD_water_) and local precipitation (δ^18^O_precipitation_ and δD_precipitation_) in Western China, and investigated influences of lake latitude, elevation, and lake water salinity on δ^18^O_water_ and δD_water_ in detail. In their results, isotope enrichment by local evaporation, of differing magnitudes depending on location, is also recorded by the Local Evaporation Line (LEL) that shifts to the right of the Local Meteoritic Water Line (LMWL) (Fig. 7c). However, the spatial distribution of lake water isotopes is in accordance with that of precipitation isotopes, with heavier δ^18^O_precipitation_ and δD_precipitation_ corresponding to more enriched δ^18^O_water_ and δD_water_ at same region in Western China (Fig. 8). Feng et al*.*^64^ concluded that δ^18^O_water_ in these lakes located in Western China is mainly controlled by δ^18^O_precipitation_ that depends on the source of water vapor, while local evaporation, lake elevation and latitude have less influence on δ^18^O_precipitation_ and δ^18^O_water_.

As changes in δ^18^O_water_ dominate the variations in δ^18^O_carb_, the spatial distribution of δ^18^O_carb_ could be inherited from that of δ^18^O_water_ in Western China. Based on Rayleigh fractionation, isotope values for precipitation become gradually depleted when water vapor climbs high mountains^65,66^. As shown in Fig. 5a,b, correlation coefficients (r) of elevation are 0.76 (*P* < 0.0001) and 0.88 (*P* < 0.0001) for carbonate and lake water, respectively, demonstrating that altitude effect is significant on the Tibetan Plateau. But δ^18^O_carb_ variances caused by the altitude effect is not significant (r = 0.78, *P* = 0.0243) at the Northwestern Xinjiang and Inner Mongolia regions where lake elevation is below 3000 m. On a worldwide scale, water vapor originates from the tropical ocean^65^. The latitude effect usually results in heavy oxygen isotope depletion of precipitation when water vapor is transported from southern to northern regions^65,67^. Although δ^18^O_water_ and δ^18^O_carb_ are negatively correlated to elevation, they are positively correlated to latitude for lakes located on the Tibetan Plateau. It indicates that the variation of δ^18^O_water_ and δ^18^O_carb_ may not be explained by the latitude effect in Tibetan Plateau. For the southern Tibetan Plateau, precipitation with negative isotope value is mainly originated from the Bay of Bengal and the Arabian Sea^68^. But the oceanic water vapor is usually blocked by the Himalayas and Tanggula Mountains and can hardly arrive northern part of the plateau^49,69^. Lakes located on the northern Tibetan Plateau are under the control of dry continental air masse with enriched heavy isotopes^64^. As a result, δ^18^O_water_ and δ^18^O_carb_ values of lakes located on the northern Tibetan Plateau are higher than these of lakes located on the southern Tibetan Plateau. Since δ^18^O_precipitation_ values provided by the westerlies and East Asia summer monsoon are different^67^, the insignificant Lat/δ^18^O_carb_ coefficient (r = 0.33, *P* = 0.4238) for lakes located in the Northwestern Xinjiang and Inner Mongolia regions could be also attributed to different vapor sources. As shown in Fig. 5e,f, the correlations between lake longitude and δ^18^O_carb_ (r = 0.41, *P* = 0.0228) and δ^18^O_water_ (r = 0.57, *P* = 0.0008) are insignificant in Western China where precipitations are originated from different water sources. It implies that there is not a continental effect that causes a general shift towards lower δ^18^O values in lake water and precipitated carbonates from east to west in Western China. Therefore, different water vapor sources contribute to the distinct spatial distribution of δ^18^O_carb_ for lakes located in different regions in Western China.

In conclusion, although disequilibrium fractionation occurs during authigenic carbonate precipitation, the δ^18^O_carb_ can be predominantly interpreted primarily as δ^18^O_water_, which in turn indirectly reflects changes in δ^18^O_precipitation_. The spatial variations of δ^18^O_carb_ in Western China are ultimately controlled by water vapor source.

In natural lacustrine settings, the temperature dependent oxygen isotope equilibrium fractionation between lacustrine carbonate and lake water can be complicated by multiple environmental controls and processes, such as ionic saturation, DIC speciation, growth rate, and other factors^70,71^. It is likely that the crystallization and precipitation of lacustrine carbonate occurs under non-equilibrium conditions^17^. As such, even though temperature is an essential factor controlling the oxygen isotopic composition of carbonate minerals, it is questionable to reconstruct water temperature using lacustrine authigenic carbonates, without a detailed discussion of whether isotopic equilibrium was attained. In contrast, our results indicate that the isotopic composition of lacustrine authigenic carbonate depends on that of host water, regardless of whether the isotope equilibrium conditions are reached. Therefore, in paleoclimate reconstructions, changes in oxygen isotopic composition of lacustrine authigenic carbonate from terminal lakes in Western China can potentially be interpreted as the oxygen isotopic composition of lake water, which in turn indirectly reflects variations of the oxygen isotopic composition of precipitation, assuming intra-annual changes in temperature were relatively small. Furthermore, the spatial distributions of lacustrine authigenic carbonate oxygen isotope values could reflect different water vapor origins in Western China.

Overall, the main conclusions we reached are: (1) Temperature is associated with a relatively small fraction of the observed variance in δ^18^O_carb_ and 1000lnα_(carb−water)_ in natural lake settings in Western China; (2) Many factors may lead to kinetic oxygen isotope fractionation during authigenic carbonate precipitation in lacustrine settings, including pH and growth rate-related effects. These factors can account for a larger fraction of the variance in δ^18^O_carb_ and 1000lnα_(carb−water)_ in these samples; (3) A positive correlation between δ^18^O_water_ and δ^18^O_carb_ is observed in the 33 lakes located in Western China. δ^18^O_water_ is the dominant factor governing δ^18^O_carb_, regardless of whether the isotope equilibrium conditions are reached during the precipitation of authigenic carbonates; (4) The spatial distribution of δ^18^O_carb_ is consistent with that of δ^18^O_water_ and δ^18^O_precipitation_, and is ultimately controlled by water vapor source in Western China; (5) Under either the presence or absence of isotope equilibrium, changes in δ^18^O_carb_ from terminal lakes in Western China can be predominantly interpreted as variations of δ^18^O_precipitaion_, instead of changes in temperature. This provides an important basis for future paleoclimatic reconstructions using the carbonate oxygen isotope proxy in lacustrine authigenic carbonates.

## Methods

### Sampling of lake surface sediment and water

In July and August 2016, surface sediment and water samples were collected from thirty-three lakes located in Western China. To ensure that the samples were not influenced by hydrological or human disturbance, samples were collected at the lake center for smaller lakes and at least 2 km away from the shore for larger lakes.

In each lake, the upper most 0.5 cm of surface sediments were collected using a stainless grab and were placed in leak proof plastic bags. At the same site, surface water samples were collected at a depth of ~ 50 cm below the water surface. Water samples were collected and stored in 500 ml high-density polyethylene (HDPE) bottles which were initially washed three times using the lake water. The bottles were completely filled with water samples and sealed with a cap secured with plastic electrical tape to avoid evaporation or any isotopic exchange with air. Sediment and water samples were kept cool in the field and were then stored at 4 °C in Capital Normal University, China.

T_MTW_ was measured once for each lake at around 2 p.m., during the warmest time of day, when the sediment and water samples were collected in the field. T_MTW_ was manually measured using a mercurial thermometer at 50 cm below the water surface in the same location of water sampling. HOBO U22 Water Temperature Pro v2 data loggers were also set at a depth of 50 cm below the water surface of each lake. Temperature data was collected at 15-min intervals over the course of one year. We returned in the following summer and successfully retrieved 12 data loggers, while the rest of the loggers were lost.

### Water chemistry and stable isotope analyses

Lake surface water samples were analyzed for K^+^, Ca^2+^, Mg^2+^, $\text{SO}^{2-}_{4}$, Cl^−^, $\text{HCO}^{-}_{3}$, and $\text{CO}^{2-}_{3}$ concentrations at the Qinghai Institute of Salt Lakes, Chinese Academy of Sciences, China. All analyses for major ions in this study followed procedures of the Qinghai Institute of Salt Lakes^72^. Cl^−^ concentrations were determined by AgNO_3_ potentiometric titration, with a precision of ± 0.1%. $\text{CO}^{2-}_{3}$ and $\text{HCO}^{-}_{3}$ concentrations were analyzed by HCl titration, with a precision of ± 0.3%. Concentrations of $\text{SO}^{2-}_{4}$ were determined by gravimetric methods through precipitation of BaSO_4_. Concentrations of K^+^ were measured by gravimetric methods through precipitation of potassium tetraphenylborate [KB(C_6_H_5_)_4_]. Ca^2+^ and Mg^2+^ concentrations were measured by ethylene diamine tetraacetic acid (EDTA) titration with errors of ± 0.5%. Na^+^ concentrations were calculated by charge balance:

9 $$\left[ {{\text{Na}}^{ + \cdot } \left] { \, = \, } \right[\left( {{\text{N}}_{{{\text{CO}}_{3}^{2 - \cdot } }} + {\text{N}}_{{{\text{HCO}}_{3}^{ - \cdot } }} + {\text{N}}_{{{\text{SO}}_{4}^{2 - \cdot } }} + {\text{N}}_{{{\text{Cl}}_{ \cdot }^{ - \cdot } }} } \right) - \left( {{\text{N}}_{{{\text{K}}_{ \cdot }^{ + \cdot } }} + {\text{N}}_{{{\text{Ca}}_{ \cdot }^{2 + \cdot } }} + {\text{N}}_{{{\text{Mg}}_{ \cdot }^{2 + \cdot } }} } \right)} \right]$$

where N represents the ionic equivalent value. The analytical precision for major cations and anions is better than ± 2%. Water salinity was calculated based on the concentrations of major aqueous ions. pH values were measured in the field during sample collection with a Mettler SevenGo2-ELK. At each lake, the probe was calibrated three times using standard pH calibration solutions (4, 6.86 and 9.18 at 25 °C). pH values of standard calibration solutions were also adjusted for measured water temperatures at the field sites. The distribution of species and carbonate SI values were calculated using the equilibrium geochemical speciation/mass transfer model PHREEQC v. 2.18.00. software program^54^ with the speciation model wateq. database.

δ^18^O_water_ and δD_water_ were conducted at the Nanjing Institute of Geography and Limnology, Chinese Academy of Sciences, China using an LGR DLT-100 Liquid Water Isotope Analyzer (Los Gatos Research, Inc., Mountain View, CA, USA). Calibration of the measurements used three internal LGR standards (δ^18^O: − 2.80‰, − 7.69‰, and − 13.10‰; δD: − 9.5‰, − 51.0‰, and − 96.4‰). δ^18^O_water_ and δD_water_ were reported relative to VSMOW. The measurement accuracy was typically better than ± 0.1‰ for δ^18^O_water_ and ± 0.5‰ for δD_water_.

### Sediment sample pretreatments

The wet surface sediment samples were soaked in deionized water for about 2 h and then wet sieved with a 350-mesh (45 μm) sieve. Materials exceeding 45-μm containing detrital mixtures and biogenic carbonates (containing primarily ostracods) were filtered out^37,73^. Only fine sieved fractions smaller than 45 μm were collected, frozen in a refrigerator overnight and then vacuum freeze-dried for 48 h using the Boyikang FD-1A-50 Freeze Dryer at approximately − 50 °C (30 Pa), until the samples were dried. Around 2 g of each sieved sediment was ground using agate mortar and pestle, and stored in a desiccator.

### Sediment X-ray powder diffraction analyses

Around 0.5 g of sediment powder were loaded into a plastic sample holder and the surface of the powder was smoothed prior to XRD measurements that were performed at the Qinghai Institute of Salt Lakes, Chinese Academy of Science, China, using a Phillips X-pert Pro X-ray diffraction with Cu K_α_ radiation (λ = 1.5406 Å). The diffraction spectral pattern was measured at a scanning rate of 2° min^-1^ for 2θ ranging from 10° to 80°. Mineral identification and semi-quantitative analyses were estimated from the bulk mineral diffractograms using the reference-intensity ratio (RIR) matrix-flushing method^74–76^ aided by the use of an automated search-match computer program X’pert HighScore Plus. The uncertainty of this semi-quantitative analysis was approximately ± 5% (1σ).

### Carbonate oxygen and carbon isotope analyses

The fine sieved sediment samples were treated with 3% H_2_O_2_ for 4 h to remove any remaining organic material. Resulting samples were collected on a 0.45 μm cellulose nitrate filter membrane and oven-dried at 40 °C. Depending on instrument sensitivity and carbonate content, the amount of sample used for isotope analyses varied between 12 and 95 mg.

The δ^18^O_carb_ and δ^13^C_carb_ were measured with a Thermo Scientific MAT 253 gas source isotope mass spectrometer at the University of California, Los Angeles, USA from 2017 to 2018. Samples were reacted with 105% phosphoric acid (ρ = 1.92 g/mL) for 20 min on a 90 °C online common phosphoric acid bath system to convert to CO_2_ gas for analyses. The liberated CO_2_ was successively passed through a dry ice/ethanol trap (− 76 °C) and a liquid nitrogen trap (− 196 °C) to remove water and other compounds. After the initial purification step, the CO_2_ was passed through silver wool to remove sulfur compounds and then passed through a Porapak Q gas chromatograph column at − 20 °C to remove any additional contaminants before being transferred into bellows of the mass spectrometer for analysis. Data were collected over 9 acquisition cycles to determine δ^13^C and δ^18^O. A high purity pre-calibrated CO_2_ tank was used as a reference gas (From 1/19/2017 to 2/21/2018: Source was Air Liquide with δ^18^O = 19.31‰ VSMOW, δ^13^C =  − 3.38‰ VPDB; after 2/21/2018: Source was Oztech with δ^18^O =  − 15.84 ‰ VPDB, δ^13^C =  − 3.64‰ VPDB), whose composition has been determined by Oztech through comparison with NBS standard gases and CO_2_ evolved by acid digestion from NBS-19 and NBS-18. At least three replicates per sample were performed.

For calcite, ^18^O/^16^O fractionation by phosphoric acid digestion at 90 °C was corrected using an acid fractionation factor of 1.00795^77^. For aragonite, an acid fractionation factor value of 1.00854 was calculated by extrapolating the relationship reported by Kim et al*.*^78^. For dolomite, we used an acid fractionation factor of 1.0093^79^. For monohydrocalcite, we used the same fractionation factor as calcite^80^. For samples that are a mixture of two or three carbonate minerals as determined by XRD, weighted acid fractionation factors were calculated.

δ^18^O_carb_ and δ^13^C_carb_ are reported on the VPDB scale. We ran NBS-19 standards. The average measured value for NBS-19 is: δ^13^C =  − 2.143 ± 0.021‰ VPDB, δ^18^O = 1.938 ± 0.012‰ VPDB (n = 6). We also ran ETH-1 to 4 as standards^81^. Carbonate standards were analyzed between every 2–3 samples and were prepared and analyzed in the same manner as samples.

Based on the stable isotope mixing model:

10 $$\delta^{{{18}}} {\text{O}}_{{{\text{carb}}}} = a*\delta^{{{18}}} {\text{O}}_{{{\text{calcite}}}} + b*\delta^{{{18}}} {\text{O}}_{{{\text{aragonite}}}} + c*\delta^{{{18}}} {\text{O}}_{{{\text{dolomite}}}} ,\left( {a + b + c = { 1}} \right)$$

where *a, b* and* c* denote the content of calcite, aragonite and dolomite, respectively. We calculated δ^18^O_carb_ using weighted fractionation factors to reduce uncertainties caused by mineral specific fractionation processes:

11 $$\delta^{18} {\text{O}}_{{{\text{carb}}}} = \delta^{18} {\text{O}}_{{{\text{carb}^{\prime}}}} + x*{\text{f}}_{{{\text{aragonite}}}} + y*{\text{f}}_{{{\text{dolomite}}}}$$

where δ^18^O_carb'_ denotes raw carbonate δ^18^O results. *x* and* y* denote the content of aragonite and dolomite, respectively. *f*_aragonite_ and *f*_dolomite_ denote the offset of δ^18^O between dolomite, aragonite and calcite at a certain water temperature in each lake^8,82^.

### Published isotopic data compilation

We synthesized δ^18^O_carb_ data including laboratory synthetic carbonates, core top lacustrine sediments and other field samples, and δ^18^O_water_ from published papers. Synthetic carbonates were included within this study if their precipitation conditions were similar to natural settings (temperatures: 5–35°C, pH: 7–10).

### Boosted regression tree analyses

BRT analyses were employed to estimate contributions of the lake water parameters to lacustrine authigenic carbonate isotopic composition. All BRT models were generated using the *gbm.step* function in the *dismo* package 1.1-4 version^83^ in R 3.6.2^84^. We used a default bag fraction of 0.5, a Gaussian error distribution, a very slow learning rate (0.0005) and a tree complexity of 3. The local polynomial regression (LOESS) curves were fitted using the *loess* function in R with span at 0.75, to validate the obtained BRT curves.

## Supplementary information

Supplementary Information.

## Data Availability

All data pertinent to this manuscript and its reported findings can be found in the manuscript itself or the associated Supplementary Information file.
