# Supplementary material for: Factors controlling the oxygen isotopic composition of lacustrine authigenic carbonates in Western China: implications for paleoclimate reconstructions
Source: Sci Rep. 2020 Oct 1;10:16370. doi: 10.1038/s41598-020-73422-4 (PMC7529879; doi:10.1038/s41598-020-73422-4)
Supplement: Supplementary file 1 — Supplementary Information. [file 41598_2020_73422_MOESM1_ESM.pdf]

Factors controlling the oxygen isotopic composition of lacustrine authigenic carbonates: Implications for paleoclimate reconstructions

Huashu Li[1], Xingqi Liu[1*], Aradhna Tripati[2,3], Shengnan Feng[1], Ben Elliott[2], Chloe Whicker[2], Alexandrea Arnold[2], Anne Marie Kelley[2]

[1] College of Resource Environment and Tourism, Capital Normal University, 100048 Beijing, P.R. China. E-mail: xqliu@niglas.ac.cn (Xingqi Liu), lihuashu525@sina.com (Huashu Li), and Clorisfsn@126.com (Shengnan Feng)
[2] Department of Earth, Planetary, and Space Sciences, Department of Atmospheric and Oceanic Sciences, Institute of the Environment and Sustainability, Center for Diverse Leadership in Science, University of California, Los Angeles, CA 90095, USA. E-mail: atripati@g.ucla.edu (Aradhna Tripati), benm.elliott@epss.ucla.edu (Ben Elliott), chloe.whicker@gmail.com (Chloe Whicker), ajarnold1@ucla.edu (Alexandrea Arnold), panter2dobey@ucla.edu (Anne Marie Kelley)
[3] European Institute of Marine Sciences (IUEM), Université de Brest, UMR 6538, Domaines Océaniques, Rue Dumont D'Urville, and IFREMER, Laboratoire Géophysique et enregistrement Sédimentaire, 29280 Plouzané, France

*Corresponding author

Xingqi Liu
College of Resource Environment and Tourism, Capital Normal University
105 West-Third-Ring North Road, Haidian District, 100048 Beijing, P.R. China
E-mail address: xqliu@niglas.ac.cn
Telephone number: +86-10-68903524 (Office)
Fax number: +86-10-68902339

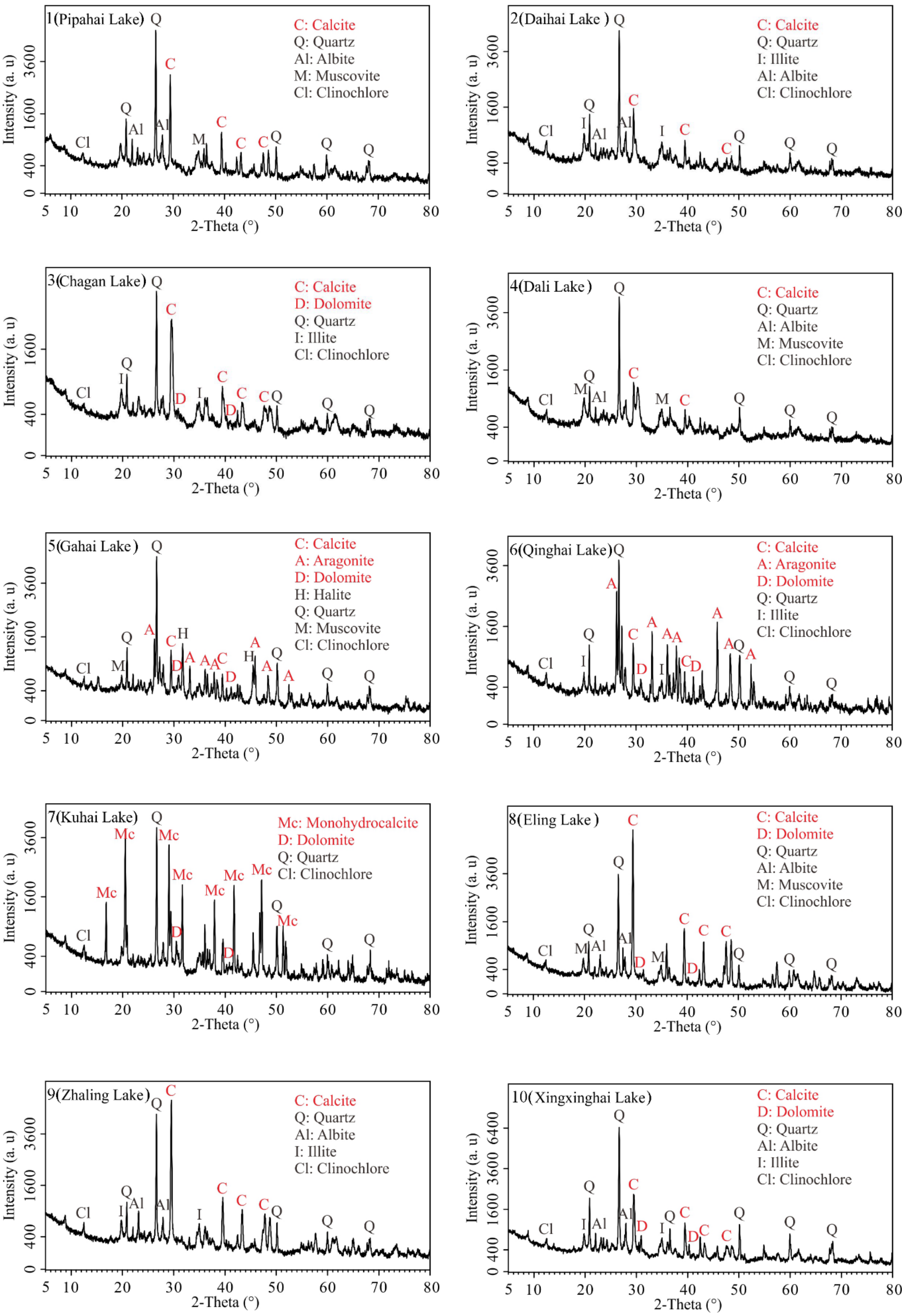
1(Pipahai Lake)
C: Calcite
Q: Quartz
Al: Albite
M: Muscovite
Cl: Clinochlore
2(Daihai Lake)
C: Calcite
Q: Quartz
I: Illite
Al: Albite
Cl: Clinochlore
3(Chagan Lake)
C: Calcite
D: Dolomite
Q: Quartz
I: Illite
Cl: Clinochlore
4(Dali Lake)
C: Calcite
Q: Quartz
Al: Albite
M: Muscovite
Cl: Clinochlore
5(Gahai Lake)
C: Calcite
A: Aragonite
D: Dolomite
H: Halite
Q: Quartz
M: Muscovite
Cl: Clinochlore
6(Qinghai Lake)
C: Calcite
A: Aragonite
D: Dolomite
Q: Quartz
I: Illite
Cl: Clinochlore
7(Kuhai Lake)
Mc: Monohydrocalcite
D: Dolomite
Q: Quartz
Cl: Clinochlore
8(Eling Lake)
C: Calcite
D: Dolomite
Q: Quartz
Al: Albite
M: Muscovite
Cl: Clinochlore
9(Zhaling Lake)
C: Calcite
Q: Quartz
Al: Albite
I: Illite
Cl: Clinochlore
10(Xingxinghai Lake)
C: Calcite
D: Dolomite
Q: Quartz
Al: Albite
I: Illite
Cl: Clinochlore
Intensity (a. u)
2-Theta (°)

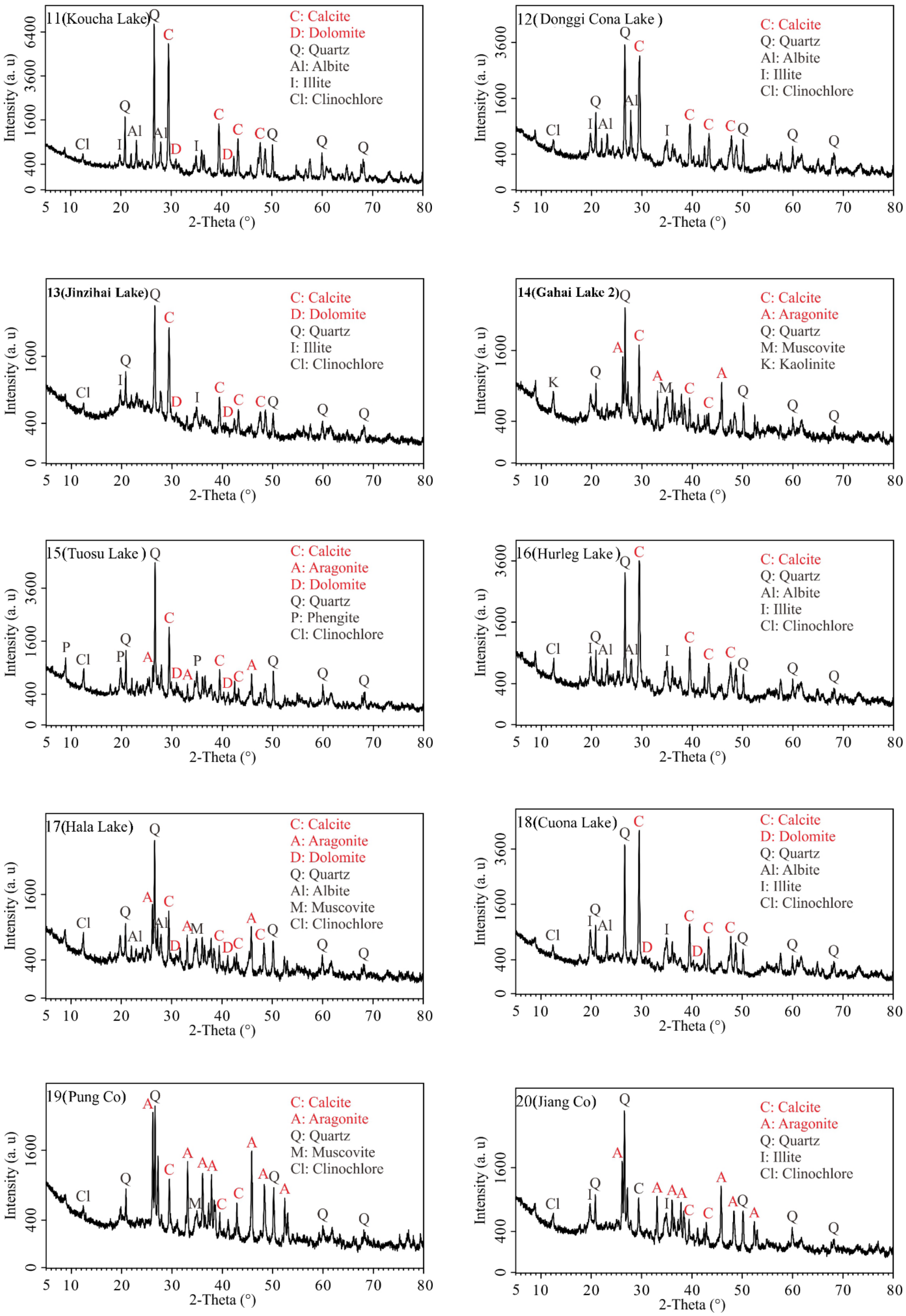
11(Koucha Lake)
C: Calcite
D: Dolomite
Q: Quartz
Al: Albite
I: Illite
Cl: Clinochlore
12(Donggi Cona Lake)
C: Calcite
Q: Quartz
Al: Albite
I: Illite
Cl: Clinochlore
13(Jinzihai Lake)
C: Calcite
D: Dolomite
Q: Quartz
I: Illite
Cl: Clinochlore
14(Gahai Lake 2)
C: Calcite
A: Aragonite
Q: Quartz
M: Muscovite
K: Kaolinite
15(Tuosu Lake)
C: Calcite
A: Aragonite
D: Dolomite
Q: Quartz
P: Phengite
Cl: Clinochlore
16(Hurleg Lake)
C: Calcite
Q: Quartz
Al: Albite
I: Illite
Cl: Clinochlore
17(Hala Lake)
C: Calcite
A: Aragonite
D: Dolomite
Q: Quartz
Al: Albite
M: Muscovite
Cl: Clinochlore
18(Cuona Lake)
C: Calcite
D: Dolomite
Q: Quartz
Al: Albite
I: Illite
Cl: Clinochlore
19(Pung Co)
C: Calcite
A: Aragonite
Q: Quartz
M: Muscovite
Cl: Clinochlore
20(Jiang Co)
C: Calcite
A: Aragonite
Q: Quartz
I: Illite
Cl: Clinochlore
Intensity (a. u)
2-Theta (°)

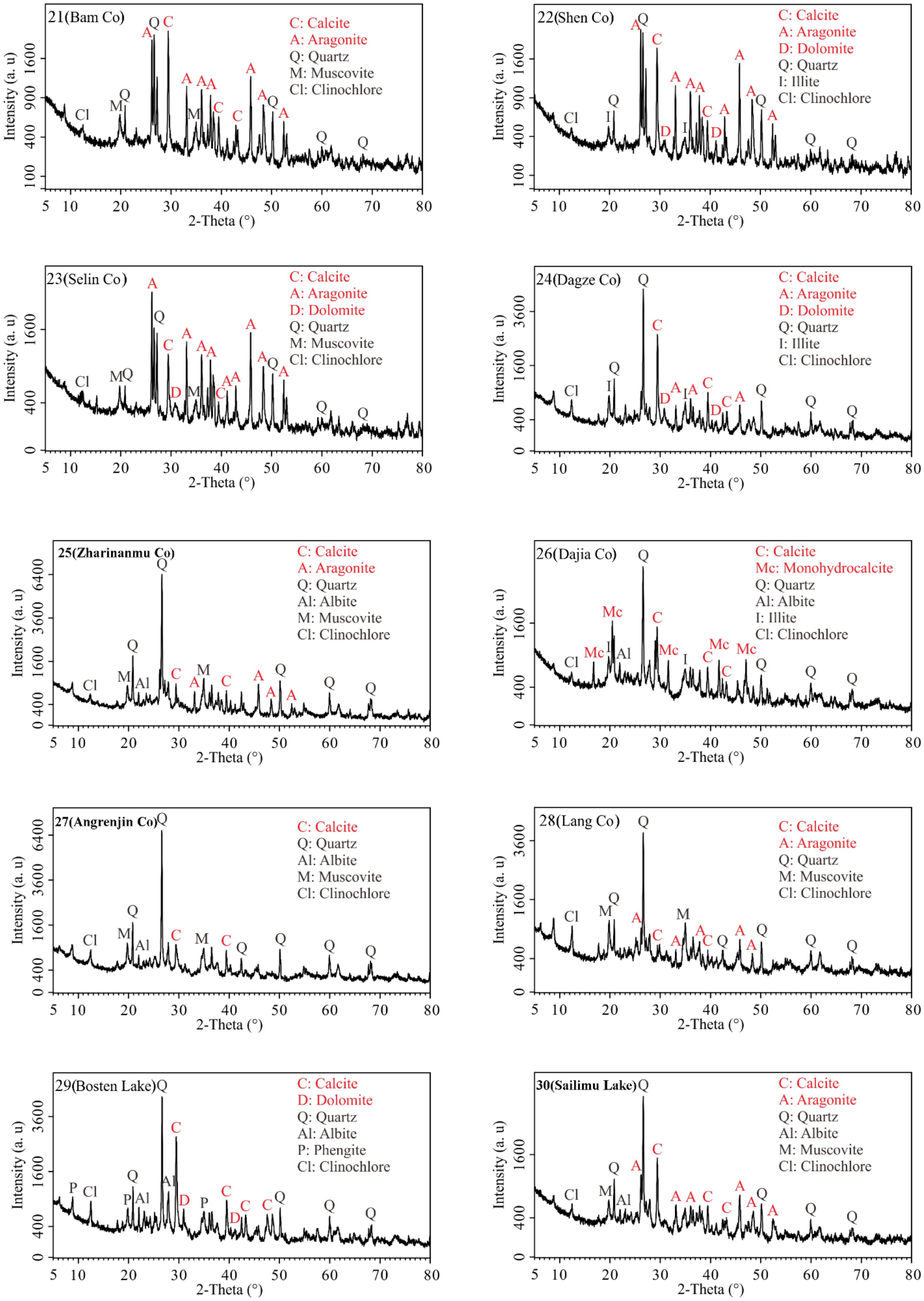
21(Bam Co)
C: Calcite
A: Aragonite
Q: Quartz
M: Muscovite
Cl: Clinochlore
22(Shen Co)
C: Calcite
A: Aragonite
D: Dolomite
Q: Quartz
I: Illite
Cl: Clinochlore
23(Selin Co)
C: Calcite
A: Aragonite
D: Dolomite
Q: Quartz
M: Muscovite
Cl: Clinochlore
24(Dagze Co)
C: Calcite
A: Aragonite
D: Dolomite
Q: Quartz
I: Illite
Cl: Clinochlore
25(Zharinanmu Co)
C: Calcite
A: Aragonite
Q: Quartz
Al: Albite
M: Muscovite
Cl: Clinochlore
26(Dajia Co)
C: Calcite
Mc: Monohydrocalcite
Q: Quartz
Al: Albite
I: Illite
Cl: Clinochlore
27(Angrenjin Co)
C: Calcite
Q: Quartz
Al: Albite
M: Muscovite
Cl: Clinochlore
28(Lang Co)
C: Calcite
A: Aragonite
Q: Quartz
M: Muscovite
Cl: Clinochlore
29(Bosten Lake)
C: Calcite
D: Dolomite
Q: Quartz
Al: Albite
P: Phengite
Cl: Clinochlore
30(Sailimu Lake)
C: Calcite
A: Aragonite
Q: Quartz
Al: Albite
M: Muscovite
Cl: Clinochlore
Intensity (a. u)
2-Theta (°)

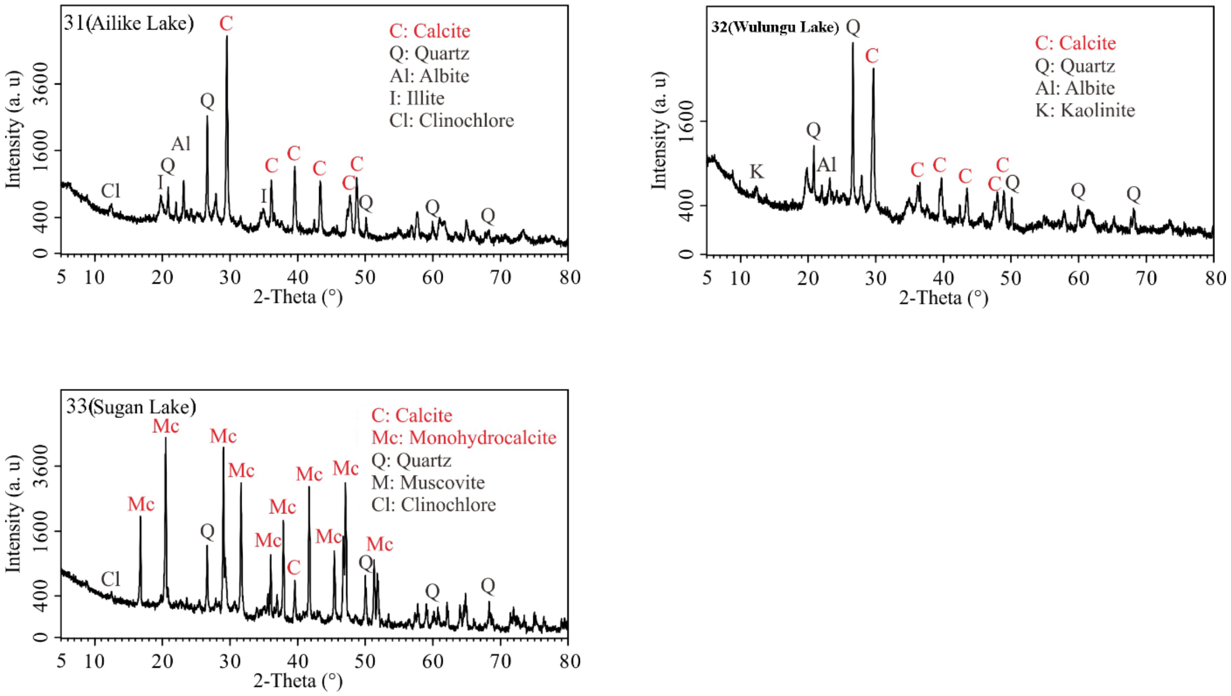

Supplementary Figure S1. XRD diffractograms of the major minerals in the lacustrine authigenic carbonate samples collected from thirty-three lakes in this study.

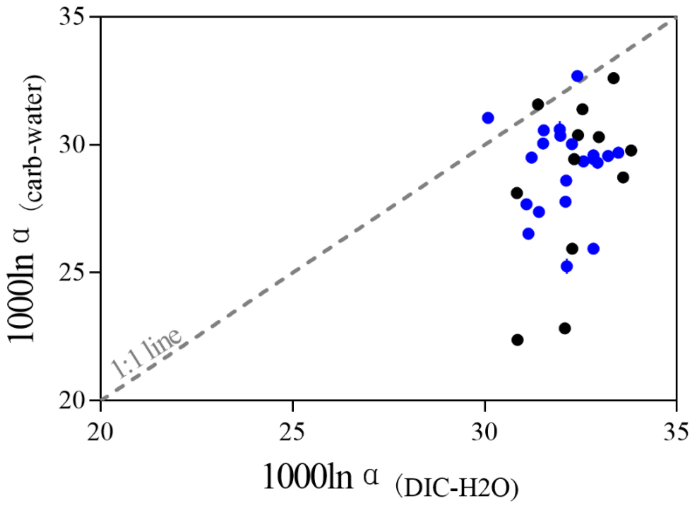

Supplementary Figure S2. Relationship between experimental 1000lnα$_{(carb - water)}$ values and expected equilibrium 1000lnα$_{(DIC - H_2O)}$ values. Points are data for thirty-three samples in this study. Black points indicate to water temperatures recorded by on-site water temperature loggers (T_{LMSW}). Blue points indicate Calculated Mean Summer Water Temperature (T_{CMSW}) for sites without data loggers, as described in the methods. Both datasets show similar results. Dashed line represents 1:1 (i.e. no difference) relationship.

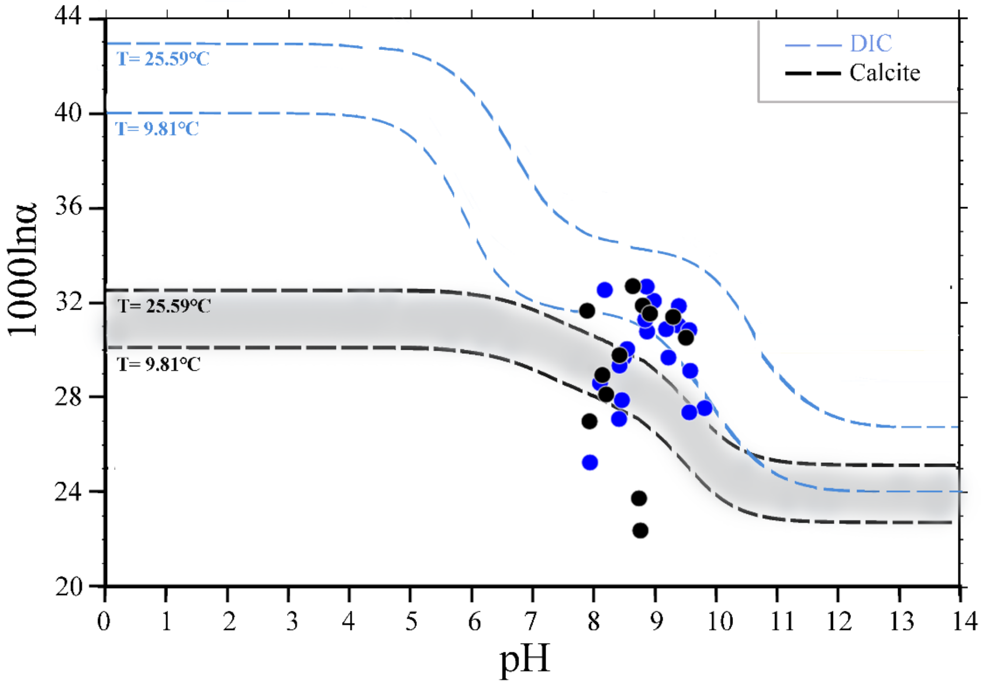

Supplementary Figure S3. Relationship between water pH and 1000lnα based on the model from Watkins *et al.*[1]. Black points refer to water temperatures directly recorded by on-site water temperature loggers (T_{LMSW}). Blue points refer to water temperatures for sites without data loggers and are calculated using the equation (1) and are reported as Calculated Mean Summer Water Temperature (T_{CMSW}). The blue dashed lines show the $1000\ln\alpha_{(DIC-water)}$ values of DIC species as a function of pH, calculated for pH values at a temperature of 9.8 °C (minimum water temperature in our study) and a temperature of 25.6 °C (maximum water temperature in our study), respectively, and using published fractionation factors[2]. The black dashed lines show $1000\ln\alpha_{(calcite-water)}$ values of calcite grown at different pH, at temperatures of 9.8 and 25.6 °C, respectively[1]. The shaded area represents the range of 'permissible' $1000\ln\alpha_{(calcite-water)}$ for calcite growth following the pH-dependent model established by Watkins *et al.*[1]. If water pH dominates the oxygen isotope fractionation between carbonate and water, most of our data points should fall in the shaded areas.

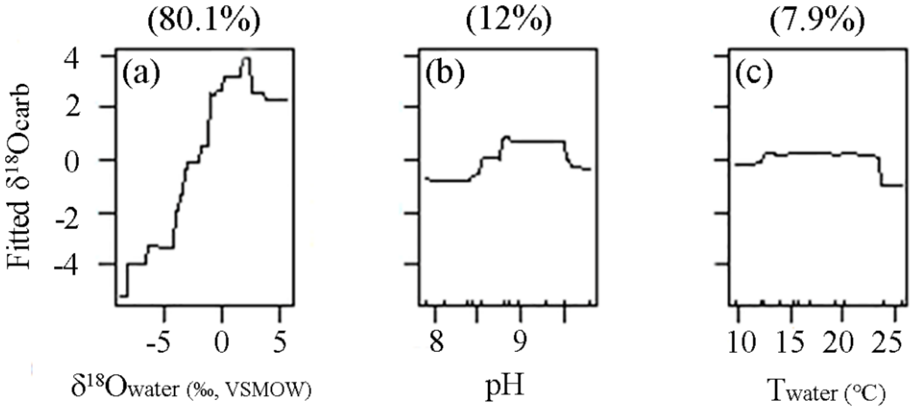

Supplementary Figure S4. Boosted regression tree (BRT) partial dependence plots showing the effect of (a) $\delta^{18}O_{water}$ on $\delta^{18}O_{carb}$; (b) pH on $\delta^{18}O_{carb}$; (c) T_{water} on $\delta^{18}O_{carb}$. Y axes are centered to have zero mean over the data distribution. The relative influence for each predictor variable is listed on the top of each graph.

No.	Lake Name	Latitude (°N)	Longitude (°E)	Elevation (m)	Area (km^2)	Depth* (m)	Logged Mean Summer Water Temperature (T_{LMSW}) (°C)	s.e.m. (1σ)	Midday Temporal Water Temperature (T_{MTW}) (°C)	s.e.m. (1σ)	Calculated Mean Summer Water Temperature (T_{CMSW}) (°C)
1	Pipahai Lake	38.85	112.21	1770	0.4	3	—	—	20.00	0.5	19.30
2	Daihai Lake	40.59	112.69	1218	86.8	7.9	23.86	0.2	22.50	0.5	—
3	Chagan Lake	43.44	115.01	1021	109	2.7	—	—	24.00	0.5	24.04
4	Dali Lake	43.26	116.51	1228	189	8.8	—	—	23.50	0.5	23.45
5	Gahai Lake	37.02	100.57	3192	45.7	8.8	15.76	0.2	18.00	0.5	—
6	Qinghai Lake	36.60	100.65	3196	4254	25	—	—	20.00	0.5	19.30
7	Kuhai Lake	35.33	99.19	4133	49	10.2	12.46	0.2	14.00	0.5	—
8	Eling Lake	35.04	97.73	4272	628	30	—	—	15.50	0.5	13.96
9	Zhaling Lake	35.01	97.35	4298	517	8.8	—	—	18.00	0.5	16.93
10	Xingxinghai Lake	34.86	98.12	4224	26.2	9.7	—	—	17.00	0.5	15.74
11	Koucha Lake	34.01	97.24	4537	17.5	6.8	12.46	0.2	16.00	0.5	—
12	Donggi Cona Lake	35.28	98.67	4092	230	29	11.34	0.2	12.00	0.5	—
13	Jinzihai Lake	36.72	97.88	2985	0.5	9.1	17.40	0.2	18.00	0.5	—
14	Gahai Lake2	37.12	97.56	2859	37	10.2	18.35	0.2	19.50	0.5	—
15	Tuosu Lake	37.16	96.98	2804	168	14.1	17.65	0.2	19.00	0.5	—
16	Hurleg Lake	37.29	96.91	2832	58.6	3.6	—	—	20.00	0.5	19.30
17	Hala Lake	38.24	97.61	4081	625	30.2	—	—	15.50	0.5	13.96
18	Cuona Lake	31.95	91.46	4592	400	20.4	—	—	17.00	0.5	15.74
19	Pung Co	31.50	91.00	4540	136	16.5	—	—	17.00	0.5	15.74
20	Jiang Co	31.53	90.81	4616	36.1	18.9	13.09	0.2	14.50	0.5	—
21	Bam Co	31.17	90.54	4575	180	70.8	12.04	0.2	13.50	0.5	—
22	Shen Co	31.01	90.51	4744	43.3	5.6	—	—	14.00	0.5	12.18
23	Selin Co	31.57	89.11	4553	1640	16.8	—	—	14.00	0.5	12.18
24	Dagze Co	31.84	87.56	4480	245	16.7	—	—	14.00	0.5	12.18
25	Zharinanmu Co	31.05	85.43	4629	1147	4.8	—	—	16.00	0.5	14.55
26	Dajia Co	29.87	85.74	5156	115	31.6	—	—	12.00	0.5	9.81
27	Angrenjin Co	29.31	87.17	4295	24.3	7.1	—	—	17.00	0.5	15.74
28	Lang Co	29.21	87.39	4303	12.1	31.8	15.30	0.2	17.50	0.5	—
29	Bosten Lake	41.86	86.78	1044	1646	4.5	—	—	23.00	0.5	22.86
30	Sailimu Lake	44.64	81.24	2078	1408	78.9	—	—	19.50	0.5	18.71
31	Ailike Lake	45.93	85.80	270	55	5.2	25.59	0.2	25.00	0.5	—
32	Wulungu Lake	47.10	87.20	482	760	5.2	—	—	22.00	0.5	21.67
33	Sugan Lake	38.88	93.91	3000	104	5	—	—	17.00	0.5	15.74

* " Depth " refers to lake water depth where we collected sediment samples.

Supplementary Table S1. Information of 33 lakes in this study.

No.	Lake Name	CO_3^{2-} (mg/L)	HCO_3^- (mg/L)	Cl^- (mg/L)	SO_4^{2-} (mg/L)	Ca_2^+ (mg/L)	Mg_2^+ (mg/L)	K^+ (mg/L)	Na^+ (mg/L)	Salinity (mg/L)	pH	$\delta^{18}O_{water}$ (‰, VSMOW)	s.d. (1σ)	δD_{water} (‰, VSMOW)	s.d. (1σ)	SI (calcite)	SI (aragonite)	SI (dolomite)
1	Pipahai Lake	0.00	246.18	46.24	49.39	34.03	27.52	11.15	48.70	463.22	7.94	2.92	0.07	-8.01	0.26	0.29	0.15	0.77
2	Daihai Lake	204.85	700.67	5209.59	222.26	47.80	218.72	16.47	3424.14	10044.50	8.76	3.78	0.05	1.92	0.01	1.22	1.07	3.47
3	Chagan Lake	37.25	624.92	69.36	34.99	22.69	48.17	16.97	198.46	1052.79	8.46	-2.19	0.01	-35.34	0.02	0.98	0.83	2.64
4	Dali Lake	1042.86	1628.58	1564.42	333.40	7.56	27.52	206.20	2402.40	7212.94	9.38	-2.77	0.04	-35.17	0.02	1.15	1.01	3.29
5	Gahai Lake	633.17	587.05	14179.96	6709.08	756.31	963.33	479.49	10131.86	34440.25	8.80	1.14	0.02	-0.67	0.15	1.92	1.77	4.21
6	Qinghai Lake	521.43	587.05	5764.46	2288.50	151.26	669.75	182.40	3903.20	14068.04	8.98	1.44	0.04	5.65	0.41	1.57	1.43	4.09
7	Kuhai Lake	297.96	435.55	8076.41	4980.36	90.76	1834.92	9.20	4430.26	20155.42	8.64	-0.85	0.06	-21.15	0.20	0.79	0.64	3.09
8	Eling Lake	18.62	189.37	77.07	21.40	30.25	25.23	2.83	61.70	426.48	8.49	-3.54	0.06	-36.01	0.35	0.6	0.44	1.31
9	Zhaling Lake	18.62	227.24	208.08	47.33	34.03	45.87	5.53	128.32	715.03	8.42	-1.58	0.06	-25.63	0.17	0.64	0.49	1.66
10	Xingxinghai Lake	18.62	227.24	127.16	22.64	22.69	38.99	4.64	90.61	552.60	8.41	-0.84	0.04	-23.94	0.15	0.47	0.32	1.41
11	Koucha Lake	0.00	170.43	80.92	8.23	30.25	25.23	2.27	36.85	354.18	8.14	-3.55	0.01	-37.19	0.00	0.21	0.06	0.51
12	Donggi Cona Lake	18.62	189.37	96.33	65.86	30.25	34.40	4.76	76.98	516.58	8.42	-3.99	0.01	-38.53	0.00	0.46	0.31	1.13
13	Jinzihai Lake	0.00	170.43	242.75	111.13	34.03	25.23	7.20	183.67	774.45	7.89	-8.60	0.05	-60.45	0.21	0	-0.15	0.12
14	Gahai Lake2	37.25	321.93	43618.79	12858.38	453.79	3945.08	417.31	26338.71	87991.23	7.93	3.49	0.02	-5.21	0.14	0.66	0.51	2.6
15	Tuosu Lake	484.19	454.49	11837.18	6651.46	30.25	1853.27	293.70	7683.00	29287.53	8.74	5.65	0.06	11.22	0.10	0.39	0.24	2.85
16	Hurleg Lake	0.00	170.43	235.05	181.10	37.82	50.46	6.50	160.55	841.91	8.10	-4.48	0.05	-35.31	0.03	0.24	0.09	0.89
17	Hala Lake	521.43	473.42	8107.24	2403.74	75.63	1082.60	168.90	4747.99	17580.96	8.87	1.45	0.08	-4.69	0.18	1.01	0.86	3.4
18	Cuona Lake	18.62	227.24	19.27	21.40	26.47	29.82	4.95	32.94	380.72	8.44	-8.82	0.08	-77.53	0.60	0.6	0.45	1.48
19	Pung Co	2327.82	1514.95	774.50	3790.84	3.78	91.75	360.58	4277.14	13141.36	9.56	-3.73	0.07	-53.04	0.32	0.67	0.52	3
20	Jiang Co	614.54	549.17	2015.25	13138.27	60.50	1376.19	447.31	5333.81	23535.05	8.92	-5.98	0.01	-67.74	0.16	0.8	0.65	3.17
21	Bam Co	1433.94	1022.59	1294.69	2905.90	7.56	110.10	326.75	3302.28	10403.81	9.51	-5.58	0.06	-65.81	0.17	0.9	0.75	3.19
22	Shen Co	2830.63	2745.86	1059.64	1393.27	3.78	48.17	365.49	4242.97	12689.80	9.58	-3.75	0.08	-53.90	0.05	0.91	0.76	3.2
23	Selin Co	577.30	624.92	1918.92	3901.97	11.34	176.61	332.21	3244.44	10787.71	9.18	-3.18	0.05	-47.05	0.07	0.6	0.45	2.59
24	Dagze Co	4916.35	1533.89	1256.16	5252.02	7.56	114.68	522.59	7133.51	20736.77	9.81	-5.45	0.02	-63.64	0.17	0.96	0.8	3.32
25	Zharinanmu Co	1433.94	416.61	1444.97	4725.17	7.56	250.01	376.40	3748.06	12402.71	9.39	-7.54	0.08	-79.12	0.08	0.35	0.2	2.46
26	Dajia Co	484.19	568.11	262.02	4564.64	15.13	38.99	68.99	2805.56	8807.63	9.22	-7.07	0.05	-79.08	0.13	0.7	0.54	1.97
27	Angrenjin Co	1769.14	1988.38	277.43	946.68	11.34	6.88	6.63	2705.19	7711.68	9.56	-4.54	0.00	-70.70	0.05	1.39	1.24	2.88
28	Lang Co	495.36	859.74	161.84	92.61	3.78	68.81	14.56	709.06	2405.75	9.30	-5.67	0.01	-78.11	0.01	0.74	0.59	3.01
29	Bosten Lake	0.00	151.50	69.36	123.48	52.94	22.94	4.48	54.37	479.06	8.18	-7.98	0.07	-54.16	0.05	0.53	0.38	1.01
30	Sailimu Lake	175.05	465.85	342.94	870.53	15.13	357.81	21.20	242.13	2490.64	8.84	-2.22	0.05	-22.72	0.17	0.68	0.53	3.01
31	Ailike Lake	0.00	340.86	231.20	80.26	30.25	27.52	9.88	224.00	943.98	8.20	-4.43	0.02	-51.11	0.14	0.64	0.5	1.61
32	Wulungu Lake	55.87	321.93	466.24	652.39	45.38	64.22	59.16	569.89	2235.08	8.54	-4.67	0.01	-49.56	0.09	0.87	0.72	2.19
33	Sugan Lake	465.56	284.05	8184.30	8413.10	90.76	1559.68	349.67	6532.90	25880.02	8.86	2.11	0.04	-3.52	0.16	0.73	0.58	2.94

Supplementary Table S2. Lake water parameters of thirty-three lakes in this study.

No.	Lake Name	Carbonate content (%)	Calcite content (%)	Monohydro-calcite content (%)	Aragonite content (%)	Dolomite content (%)	$\delta^{18}O_{carb}$ (‰, VPDB)	s.e.m. (1σ)	1000lnα (carb-water)	1000lnα (DIC − H_2O)	$\delta^{13}C_{carb}$ (‰, VPDB)	s.e.m. (1σ)
1	Pipahai Lake	17	17				-2.66	0.31	25.25	32.14	1.72	0.12
2	Daihai Lake	8	8				-4.61	0.09	22.38	30.85	-4.06	0.04
3	Chagan Lake	24	23			1	-5.25	0.11	27.68	31.09	-2.35	0.00
4	Dali Lake	7	7				-2.53	0.10	31.06	30.09	0.96	0.03
5	Gahai Lake	24	5		17	2	0.58	0.27	30.38	32.43	2.39	0.21
6	Qinghai Lake	45	5		38	2	1.06	0.01	30.57	31.54	2.92	0.00
7	Kuhai Lake	50	3	46		1	0.81	0.05	32.60	33.35	2.08	0.16
8	Eling Lake	40	39			1	-4.73	0.07	29.57	33.21	-0.97	0.01
9	Zhaling Lake	26	26				-3.04	0.16	29.36	32.57	0.53	0.03
10	Xingxinghai	9	7			2	-5.64	0.01	25.94	32.83	-0.13	0.04
11	Koucha Lake	24	23			1	-5.56	0.03	28.73	33.60	4.59	0.06
12	Donggei Cona	18	18				-4.97	0.06	29.78	33.81	1.54	0.07
13	Jinzihai Lake	18	17			1	-7.88	0.07	31.39	32.55	-1.39	0.06
14	Gahai Lake2	32	10		22		-1.44	0.11	25.94	32.28	1.54	0.04
15	Tuosu Lake	21	11		9	1	-2.36	0.02	22.83	32.09	1.70	0.02
16	Hurleg Lake	22	22				-6.57	0.05	28.61	32.12	2.39	0.01
17	Har Lake	23	5		17	1	-0.02	0.06	29.44	32.84	3.88	0.03
18	Cuona Lake	23	22			1	-9.83	0.18	29.59	32.83	2.16	0.07
19	Pung Co	49	6		43		-4.98	0.13	29.51	31.22	4.84	0.10
20	Jiang Co	27	5		22		-6.37	0.16	30.31	32.97	3.24	0.04
21	Bam Co	52	15		37		-6.84	0.05	29.44	32.33	3.19	0.01
22	Shenco	53	11		40	2	-6.67	0.03	27.78	32.10	3.94	0.01
23	Selin Co	56	7		45	4	-4.63	0.14	29.31	32.94	4.97	0.09
24	Dagze Co	25	13		10	2	-9.53	0.11	26.53	31.14	1.98	0.06
25	Zharinanmu	18	3		15		-7.61	0.41	30.61	31.95	4.30	0.22
26	Dajia Co	24	8	16			-8.04	0.06	29.69	33.48	3.93	0.05
27	Angrenjin Co	4	4				-7.83	0.11	27.38	31.41	0.49	0.05
28	Lang Co	11	1		10		-6.36	0.03	30.03	32.27	2.93	0.04
29	Bosten Lake	21	17			4	-7.09	0.05	31.58	31.39	-0.92	0.01
30	Sailimu Lake	23	9		14		-2.69	0.21	30.36	31.97	2.70	0.06
31	Ailike Lake	52	52				-7.00	0.08	28.12	30.84	-3.07	0.02
32	Wulungu	27	27				-5.36	0.06	30.05	31.52	0.79	0.05
33	Sugan Lake	73	4	69			3.77	0.14	32.69	32.41	0.80	0.01

Supplementary Table S3. Lake surface carbonate analyses results.

No.	Section	Carbonate species	Sample Account	$\delta^{18}O_{carb}$ (‰, VPDB)	$\delta^{18}O_{water}$ (‰, VSMOW)	Water temperature (℃)	pH	Fractionation condition	Reference
1	Laboratory synthetic experiments	Calcite	26	−13.48 ~ −4.14	−8.30 ~ −7.74	10, 25	7.50 ~ 8.50	Equilibrium and disequilibrium	[3]
2		Calcite	4	−11.37 ~ −10.31	−7.88 ~ −7.37	23, 33	−	Equilibrium	[4]
3		Calcite	16	−13.72 ~ −7.30	−9.59	5, 25	7.50 ~ 9.40	Equilibrium	[5]
4		Calcite	3	−11.12 ~ −9.80	−7.24 ~ −9.50	14.5, 33	7.40	Equilibrium	[6]
5		Witherite	12	−13.14 ~ −7.47	−8.30 ~ −7.80	10, 25	7.50 ~ 8.50	Equilibrium and disequilibrium	[3]
6		Witherite	7	−11.13 ~ −2,71	−5.33 ~ −4.90	15, 25	6.94 ~ 9.68	Equilibrium	[2]
7		Aragonite	57	−10.78 ~ −8.40	−6.77 ~ −12.19	5, 10, 25	7.52 ~	Equilibrium	[7]
8		Calcite and Aragonite	15	−8.64 ~ −7.66	−5.81~ −6.27	25	7.00	Equilibrium	[8]
9		Calcite and aragonite mixture	31	−14.95 ~ −7.61	−12.24 ~ −4.66	25	−	Equilibrium	[9]
10		Calcite and aragonite mixture	7	−4.49 ~ −4.45	−5.40 ~ −5.90	25	8.02 ~ 9.80	Equilibrium and disequilibrium	[10]
11	Field samples	Tufa calcite	2	−10.78 ~ −14.76	−8.10 ~ −11.50	33.8, 36	7.70	−	[11]
12		Tufa calcite	13	−9.3 0~ −5.39	−10.70 ~ −6.50	9.5 ~ 33.8	6.63 ~ 8.94	−	[12]
13		Tufa calcite	27	−8.20 ~ −5.97	−8.99 ~ −7.63	5.6 ~ 16	8.22 ~ 8.45	−	[6]
14		Tufa calcite	3	−3.90 ~ −1.80	−2.70 ~ −1.30	−	−	−	[13]
15		Stalagmite calcite	4	−7.47 ~ −6.61	−9.00 ~ −8.50	9.5, 10.5	−	−	[11]
16		Stalagmite calcite	130	−5.93 ~ −4.08	−4.60 ~ −3.90	14.9 ~ 22.6	8.30	Equilibrium and disequilibrium	[14]
17		Stalagmite calcite	3	−5.00 ~ −3.13	−6.41 ~ −6.17	11.38 ~ 12.49	−	−	[15]
18		Stalagmite calcite	10	−10.08 ~ −6.38	−7.36 ~ −6.77	16.5	−	−	[16]
19		Speleothem calcite	4	−9.71 ~ −8.74	−10.30 ~ −8.10	9.8, 28.5	7.60	−	[11]
20		Speleothem calcite	44	−14.70 ~ −8.90	−9.50	10	−	−	[17]
21		Devils Hole vein calcite	1	−15.83	−13.54	33.7	7.40	Equilibrium	[18]
22		Laghetto Basso calcite	1	−4.48	−7.39	7.9	8.20	Equilibrium	[19]
23	Lake surface sediments	Modeled carbonate	30	−5.78 ~ −0.23	−5.89 ~ −0.58	7.05 ~ 8.85	−	Equilibrium	[20]
24		Lake bulk carbonate	58	−17.56 ~ 6.65	−19.90 ~ 5.57	3.0 ~ 16.3	−	−	[13,21-33]

Supplementary Table S4. Compilation of published data.

References

1 Watkins, J. M., Hunt, J. D., Ryerson, F. J. & DePaolo, D. J. The influence of temperature, pH, and growth rate on the $\delta^{18}O$ composition of inorganically precipitated calcite. *Earth and Planetary Science Letters* **404**, 332-343 (2014).

2 Beck, W. C., Grossman, E. L. & Morse, J. W. Experimental studies of oxygen isotope fractionation in the carbonic acid system at 15, 25, and 40℃. *Geochimica et Cosmochimica Acta* **69**, 3493-3503 (2005).

3 Kim, S. T. & O'Neil, J. R. Equilibrium and nonequilibrium oxygen isotope effects in synthetic carbonates. *Geochimica et cosmochimica acta* **61**, 3461-3475 (1997).

4 Ghosh, P. *et al.* ^{13}C–^{18}O bonds in carbonate minerals: a new kind of paleothermometer. *Geochimica et Cosmochimica Acta* **70**, 1439-1456 (2006).

5 Dietzel, M., Tang, J., Leis, A. & Köhler, S. J. Oxygen isotopic fractionation during inorganic calcite precipitation—Effects of temperature, precipitation rate and pH. *Chemical Geology* **268**, 107-115 (2009).

6 Kato, H. *et al.* Seasonal temperature changes obtained from carbonate clumped isotopes of annually laminated tufas from Japan: Discrepancy between natural and synthetic calcites. *Geochimica et Cosmochimica Acta* **244**, 548-564 (2019).

7 Kim, S. T., O'Neil, J. R., Hillaire Marcel, C. & Mucci, A. Oxygen isotope fractionation between synthetic aragonite and water: influence of temperature and Mg^{2+} concentration. *Geochimica et Cosmochimica Acta* **71**, 4704-4715 (2007).

8 Tarutani, T., Clayton, R. N. & Mayeda, T. K. The effect of polymorphism and magnesium substitution on oxygen isotope fractionation between calcium carbonate and water. *Geochimica et Cosmochimica Acta* **33**, 987-996 (1969).

9 Zhou, G. T. & Zheng, Y. F. An experimental study of oxygen isotope fractionation between inorganically precipitated aragonite and water at low temperatures. *Geochimica et Cosmochimica Acta* **67**, 387-399 (2003).

10 Guo, Y., Deng, W. & Wei, G. Kinetic effects during the experimental transition of aragonite to calcite in aqueous solution: Insights from clumped and oxygen isotope signatures. *Geochimica et Cosmochimica Acta* **248**, 210-230 (2019).

11 Demény, A., Kele, S. & Siklósy, Z. Empirical equations for the temperature dependence of calcite-water oxygen isotope fractionation from 10 to 70° C. *Rapid Communications in Mass Spectrometry* **24**, 3521-3526 (2010).

12 Kele, S. *et al.* Temperature dependence of oxygen-and clumped isotope fractionation in carbonates: a study of travertines and tufas in the 6–95 C temperature range. *Geochimica et Cosmochimica Acta* **168**, 172-192 (2015).

13 Huntington, K., Wernicke, B. & Eiler, J. Influence of climate change and uplift on Colorado Plateau paleotemperatures from carbonate clumped isotope thermometry. *Tectonics* **29** (2010).

14 Feng, W., Banner, J. L., Guilfoyle, A. L., Musgrove, M. & James, E. W. Oxygen isotopic fractionation between drip water and speleothem calcite: A 10-year monitoring study, central Texas, USA. *Chemical Geology* **304**, 53-67 (2012).

15 Labuhn, I. *et al.* A high-resolution fluid inclusion $\delta^{18}O$ record from a stalagmite in SW France: modern calibration and comparison with multiple proxies. *Quaternary Science Reviews* **110**, 152-165 (2015).

16 Li, H. C. *et al.* The $\delta^{18}O$ and $\delta^{13}C$ records in an aragonite stalagmite from Furong Cave, Chongqing, China: A-2000-year record of monsoonal climate. *Journal of Asian Earth Sciences* **40**, 1121-1130 (2011).

17 Demény, A. *et al.* Recrystallization-induced oxygen isotope changes in inclusion-hosted water of speleothems–Paleoclimatological implications. *Quaternary international* **415**, 25-32 (2016).

18 Coplen, T. B. Calibration of the calcite–water oxygen-isotope geothermometer at Devils Hole, Nevada, a natural laboratory. *Geochimica et Cosmochimica Acta* **71**, 3948-3957 (2007).

19 Daëron, M. *et al.* Most Earth-surface calcites precipitate out of isotopic equilibrium. *Nature communications* **10**, 429 (2019).

20 Steinman, B. A. *et al.* Isotopic and hydrologic responses of small, closed lakes to climate variability: Comparison of measured and modeled lake level and sediment core oxygen isotope records. *Geochimica et Cosmochimica Acta* **105**, 455-471 (2013).

21 Clegg, B. F. & Hu, F. S. An oxygen-isotope record of Holocene climate change in the south-central Brooks Range, Alaska. *Quaternary Science Reviews* **29**, 928-939 (2010).

22 Anderson, L., Abbott, M. B., Finney, B. P. & Burns, S. J. Regional atmospheric circulation change in the North Pacific during the Holocene inferred from lacustrine carbonate oxygen isotopes, Yukon Territory, Canada. *Quaternary Research* **64**, 21-35 (2005).

23 Tian, J., Nelson, D. M. & Hu, F. S. Possible linkages of late-Holocene drought in the North American midcontinent to Pacific Decadal Oscillation and solar activity. *Geophysical Research Letters* **33** (2006).

24 Bird, B. W., Wilson, J. J., Gilhooly III, W. P., Steinman, B. A. & Stamps, L. Midcontinental Native American population dynamics and late Holocene hydroclimate extremes. *Scientific reports* **7**, 41628 (2017).

25 Anderson, L. Rocky Mountain hydroclimate: Holocene variability and the role of insolation, ENSO, and the North American Monsoon. *Global and Planetary Change* **92-93**, 198-208, doi:10.1016/j.gloplacha.2012.05.012 (2012).

26 Anderson, L. Holocene record of precipitation seasonality from lake calcite $\delta^{18}O$ in the central Rocky Mountains, United States. *Geology* **39**, 211-214 (2011).

27 Bird, B. W. *et al.* A 2,300-year-long annually resolved record of the South American summer monsoon from the Peruvian Andes. *Proceedings of the National Academy of Sciences* **108**, 8583-8588 (2011).

28 Liu, W., Li, X., Zhang, L., An, Z. & Xu, L. Evaluation of oxygen isotopes in carbonate as an indicator of lake evolution in arid areas: the modern Qinghai Lake, Qinghai–Tibet Plateau. *Chemical Geology* **268**, 126-136 (2009).

29 Finkenbinder, M. S., Abbott, M. B. & Steinman, B. A. Holocene climate change in Newfoundland reconstructed using oxygen isotope analysis of lake sediment cores. *Global and Planetary Change* **143**, 251-261 (2016).

30 Kluge, T., Affek, H. P., Dublyansky, Y. & Spötl, C. Devils Hole paleotemperatures and implications for oxygen isotope equilibrium fractionation. *Earth and Planetary Science Letters* **400**, 251-260 (2014).

31 Morrill, C. *et al.* Holocene variations in the Asian monsoon inferred from the geochemistry of lake sediments in central Tibet. *Quaternary Research* **65**, 232-243 (2006).

32 Hodell, D. A. *et al.* Paleoclimate of southwestern China for the past 50,000 yr inferred from lake sediment records. *Quaternary Research* **52**, 369-380 (1999).

33 Sun, Q. *et al.* An oxygen isotope record from Lake Xiarinur in Inner Mongolia since the last deglaciation and its implication for tropical monsoon change. *Global and planetary change* **163**, 109-117 (2018).
